# Providing a framework for seagrass mapping in United States coastal ecosystems using high spatial resolution satellite imagery

**DOI:** 10.1016/j.jenvman.2023.117669

**Published:** 2023-03-24

**Authors:** Megan M. Coffer, David D. Graybill, Peter J. Whitman, Blake A. Schaeffer, Wilson B. Salls, Richard C. Zimmerman, Victoria Hill, Marie Cindy Lebrasse, Jiang Li, Darryl J. Keith, James Kaldy, Phil Colarusso, Gary Raulerson, David Ward, W. Judson Kenworthy

**Affiliations:** a Oak Ridge Institute for Science and Education, U.S. Environmental Protection Agency, Office of Research and Development, Durham, NC, USA; b Global Science & Technology, Inc., Greenbelt, MD, USA; c U.S. Environmental Protection Agency, Office of Research and Development, Durham, NC, USA; d Department of Earth & Ocean Sciences, Old Dominion University, Norfolk, VA, USA; e Department of Marine, Earth and Atmospheric Sciences, North Carolina State University, Raleigh, NC, USA; f Department of Electrical and Computer Engineering, Old Dominion University, Norfolk, VA, USA; g U.S. Environmental Protection Agency, Office of Research and Development, Narragansett, RI, USA; h U.S. Environmental Protection Agency, Office of Research and Development, Newport, OR, USA; i U.S. Environmental Protection Agency, Region 1, Boston, MA, USA; j Largo, FL, USA; k U.S. Geological Survey, Alaska Science Center, Anchorage, AK, USA; l Department of Biology and Marine Biology, University of North Carolina, Wilmington, NC, USA

**Keywords:** Coastal monitoring, Seagrass, WorldView-2, WorldView-3, Satellite remote sensing, Image classification

## Abstract

Seagrasses have been widely recognized for their ecosystem services, but traditional seagrass monitoring approaches emphasizing ground and aerial observations are costly, time-consuming, and lack standardization across datasets. This study leveraged satellite imagery from Maxar’s WorldView-2 and WorldView-3 high spatial resolution, commercial satellite platforms to provide a consistent classification approach for monitoring seagrass at eleven study areas across the continental United States, representing geographically, ecologically, and climatically diverse regions. A single satellite image was selected at each of the eleven study areas to correspond temporally to reference data representing seagrass coverage and was classified into four general classes: land, seagrass, no seagrass, and no data. Satellite-derived seagrass coverage was then compared to reference data using either balanced agreement, the Mann-Whitney U test, or the Kruskal-Wallis test, depending on the format of the reference data used for comparison. Balanced agreement ranged from 58% to 86%, with better agreement between reference- and satellite-indicated seagrass absence (specificity ranged from 88% to 100%) than between reference- and satellite-indicated seagrass presence (sensitivity ranged from 17% to 73%). Results of the Mann- Whitney U and Kruskal-Wallis tests demonstrated that satellite-indicated seagrass percentage cover had moderate to large correlations with reference-indicated seagrass percentage cover, indicative of moderate to strong agreement between datasets. Satellite classification performed best in areas of dense, continuous seagrass compared to areas of sparse, discontinuous seagrass and provided a suitable spatial representation of seagrass distribution within each study area. This study demonstrates that the same methods can be applied across scenes spanning varying seagrass bioregions, atmospheric conditions, and optical water types, which is a significant step toward developing a consistent, operational approach for mapping seagrass coverage at the national and global scales. Accompanying this manuscript are instructional videos describing the processing workflow, including data acquisition, data processing, and satellite image classification. These instructional videos may serve as a management tool to complement field- and aerial-based mapping efforts for monitoring seagrass ecosystems.

## 1. Introduction

Seagrasses are the only fully submerged marine angiosperms, having evolved from terrestrial plants approximately 100 million years ago. Seagrasses offer a variety of ecosystem services, detailed at length in Cullen-Unsworth and Unsworth (2013) and summarized here. Seagrasses promote sedimentation and reduce shoreline erosion (Christianen et al., 2013) by reducing wave action (Fonseca and Cahalan, 1992); provide habitats (Bertelli and Unsworth, 2014) and food resources (Prado et al., 2008) for fish, marine megafauna (Sievers et al., 2019), and invertebrates (Beck et al., 2001); mitigate ocean acidification (Hendriks et al., 2014; Koweek et al., 2018); and both store and filter nutrients, including carbon (Fourqurean et al., 2012), nitrogen, and phosphorus (Saderne et al., 2020). There are approximately 70 known species of seagrasses found along the shores of every continent except Antarctica, ten species of which are at an elevated risk of extinction (Short et al., 2011). Effective monitoring is critical for managing and protecting seagrasses and their ecosystem services (Bell et al., 2007; Duarte, 2002; Neckles et al., 2012).

The United States has approximately 55,000 km of marine coastline (U.S. Census Bureau, 2015), 20% of which has been mapped for seagrass coverage, primarily in the Chesapeake Bay and along the coasts of Florida, North Carolina, and New England (CEC, 2021). Only a few coastal locations in the United States conduct regular seagrass mapping. The Virginia Institute of Marine Science (VIMS) has mapped much of the Chesapeake Bay via aerial imagery annually since 1984. Seagrasses in North Carolina are monitored under the auspices of the Albemarle Pamlico National Estuary Partnership, with much of the North Carolina coast mapped in 2006 through 2007 and in 2013 (Field et al., 2021). The Washington State Department of Natural Resources has produced near-annual maps of portions of the Puget Sound since 2000 (PSEMP, 2015). In Florida, both the Indian River Lagoon and Florida’s Suncoast are mapped every two years and Florida’s Springs Coast is mapped every four years. The Massachusetts Department of Environmental Protection (MassDEP) has taken a multi-phase approach to mapping eelgrass along the Massachusetts coast since 1995 (Costello and Kenworthy, 2011). The Maine Department of Marine Resources mapped seagrass in 1997, 2010, and is currently working to update these mapping efforts as early as 2023.

Across the United States, several policies are in place to manage coastal seagrass habitats, with a particular emphasis on seagrass protection and monitoring. Seagrass are considered a wetland under Section 404 of the Clean Water Act, which regulates the discharge of dredged and fill material into United States waters (33 U.S.C. § 1323). At the state level, legislation in Texas (TX Parks & Wild. § 66.024, 2013), New York (NY Env Cons L §13–0705, 2019), Washington (WAC 220-660-350), and Florida (Fla. Stat. § 253.04, 2012) protects the destruction of seagrass habitats, and California has a policy requiring seagrass monitoring (NOAA Fisheries, 2014). The Chesapeake Bay, whose watershed encompasses parts of six states, requires annual surveys of its seagrasses (33 U.S.C. § 1267). Several states and tribes have adopted wetland protection programs, which include protections for seagrass ecosystems (ELI, 2007a, 2007b, 2006; Thomas et al., 2005), and many federally designated estuaries included in the U.S. Environmental Protection Agency’s National Estuary Program have supported seagrass monitoring and restoration efforts (Field et al., 2021; U.S. EPA, 2005). Additionally, eelgrass has been selected as one of twenty-five vital signs used in Washington State to track progress in the restoration and recovery of Puget Sound (Puget Sound Partnership, 2019). Yet, a recent review found that global management efforts are currently failing to adequately protect seagrass meadows (Griffiths et al., 2020). One of the five priorities recommended for improved management efforts was consistent monitoring of seagrass abundance and distribution across the submarine landscape.

Seagrass distributions have been mapped using ground-based field observations, towed underwater videography, acoustic technology, photointerpretation of aerial imagery, or some combination of these methods. Additional methods for mapping seagrass coverage have been established that rely on satellite platforms (Veettil et al., 2020), which can complement traditional seagrass mapping efforts. Satellite platforms benefit from frequent image acquisition and repeatability across time and space; additionally, satellite imagery can be cost effective for end users compared to traditional seagrass mapping methods, particularly across large spatial and temporal scales (Coffer et al., 2020; Dekker et al., 2006; Kohlus et al., 2020). Recent advancements in satellite technology have resulted in platforms that provide an unmatched combination of temporal and spatial resolution, which is ideal for mapping seagrass (Phinn et al., 2008), particularly in heterogeneous ecosystems where seagrass patch size is small (Hill et al., 2014).

Several multispectral satellite platforms now provide sufficiently high spatial resolution suitable for mapping seagrass coverage. The European Space Agency’s Sentinel-2 satellite platforms offer up to 10-m spatial resolution and up to a 5-day revisit period at the equator (Traganos et al., 2022a, 2022b). Planet’s RapidEye satellite constellation offered between 5- and 6.5-m spatial resolution and a 5.5-day revisit period before being decommissioned in 2020 (Coffer et al., 2020; Traganos and Reinartz, 2018), and their PlanetScope satellite constellation offers 3-m spatial resolution and daily revisit frequency (Wicaksono and Lazuardi, 2018). Maxar Technologies’ WorldView-2 satellite offers 1.84-m spatial resolution and WorldView-3 offers 1.24-m spatial resolution; both platforms have a daily revisit frequency, but are tasked satellites, meaning data collection is neither continuous nor standardized (Baumstark et al., 2016; Coffer et al., 2020; Lebrasse et al., 2022; Roelfsema et al., 2014). Recently, the Chesapeake Bay Program partnership, working through its Submerged Aquatic Vegetation Workgroup, the Scientific, Technical Assessment and Reporting Team, and Chesapeake Bay Scientific Technical Advisory Committee, began exploring the possibility of complementing their aerial photointerpretation efforts with satellite image classifications to reduce costs associated with continued monitoring of the Chesapeake Bay (Landry et al., 2021).

Studies leveraging satellite remote sensing for seagrass mapping typically focus on a single study area or satellite image (*e.g.*, Meyer and Pu, 2012; Traganos and Reinartz, 2017; Zoffoli et al., 2020). This study leveraged high spatial resolution, commercial satellite data from Maxar’s WorldView-2 and WorldView-3 platforms to classify seagrass distributions at eleven coastal study areas across the continental United States, making it one of the the first studies to apply consistent methods across multiple study areas and satellite images. Our goal is to define a uniform, operational framework for mapping seagrass coverage from satellite imagery that can be applied at local, national, and even global scales. Accompanying this manuscript are instructional videos describing the processing workflow applied at each study area, including data acquisition, data processing, and satellite image classification of seagrass presence and absence. These instructional videos reduce the disconnect between technical staff and management that commonly prevents satellite data from being used for water quality management (Schaeffer et al., 2013). This manuscript and the accompanying instructional videos can be used as a complementary tool for improving efficiency in monitoring seagrass coverage and can assist stakeholders in management decision making.

This study advances satellite remote sensing of seagrass ecosystems to the second validation stage outlined by the National Aeronautics and Space Administration (NASA) in their definition of data maturity levels (NASA, 2022a). Stage 1 Validation describes when a product’s performance has been assessed against field programs at a few locations. Stage 2 Validation describes when a product’s performance has been assessed against field programs over additional locations and time periods, demonstrating spatial and temporal consistency over globally representative locations. While only locations within the United States are analyzed here, the study areas represent three of six global seagrass bioregions and span a range of climate regions, optical water types, atmospheric conditions, and seagrass species.

## 2. Data and methods

### 2.1. Study areas

**Fig. 1.**
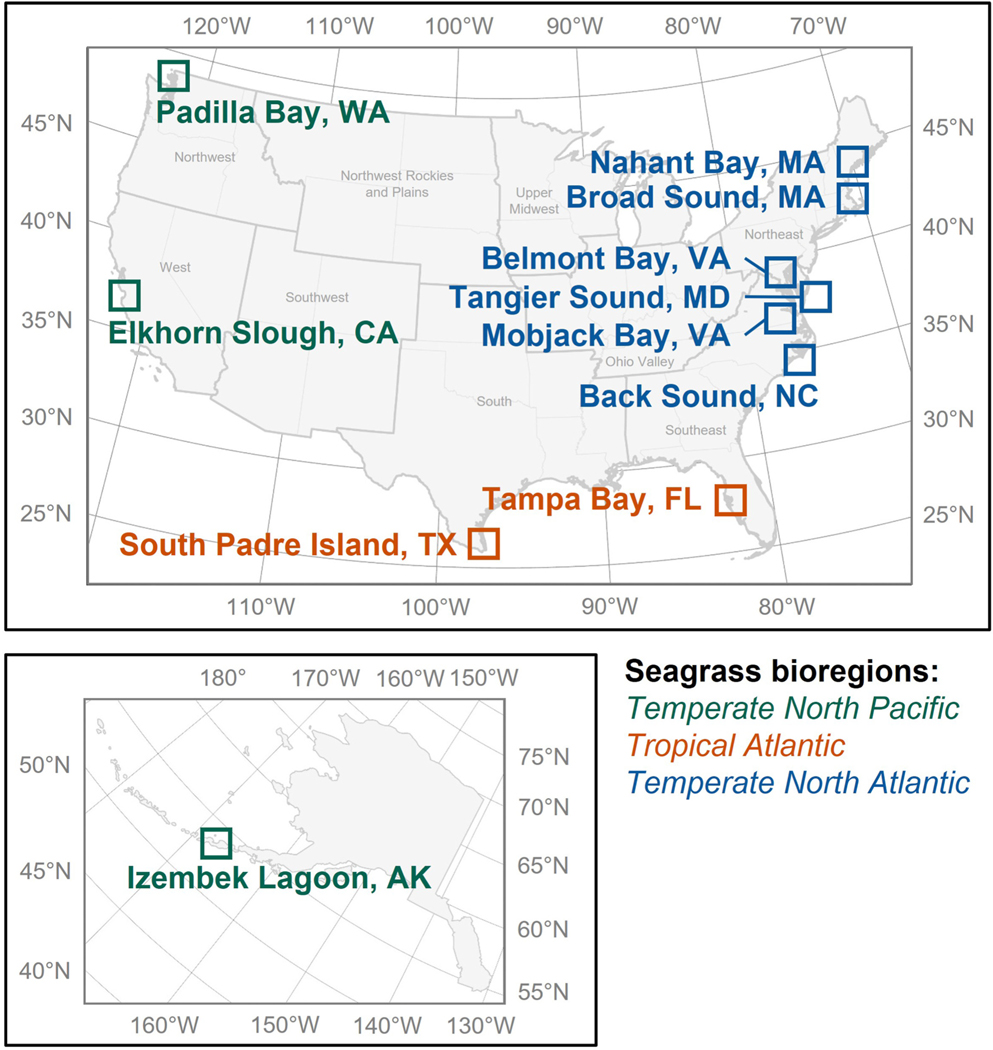
Map of the study areas. States are grouped by their climate region (gray text) as defined by Karl and Koss (1984). Each study area is also colored by its global seagrass bioregion as defined by Short et al. (2007).

Eleven study areas were chosen across the continental United States, representing geographically, ecologically, and climatically diverse regions (Fig. 1; Table S1). Details of each study area are provided in Supplemental Text S1. Each study area is characterized ecologically by its global seagrass bioregion and climatically by its United States climate region. Six global seagrass bioregions were defined by Short et al. (2007) to represent species assemblages, species distributional ranges, and tropical and temperate influences. Three of these global seagrass bioregions—Temperate North Pacific, Tropical Atlantic, and Temperate North Atlantic—include United States coastal waters. Nine climate regions were defined across the conterminous United States by The National Centers for Environmental Information (Karl and Koss, 1984) to represent climatically consistent states. Five of these climate regions—Northwest, West, South, Southeast, and Northeast—include coastal states. In addition to ecological and climatic differences across study areas, cloud cover, water column properties, and characteristics of the littoral zone varied, representing a range of optical complexities (Conmy et al., 2017).

### 2.2. Reference data delineating seagrass coverage

A combination of field survey results, observations from towed underwater cameras, and both aerial and satellite photointerpretations were used to compare satellite-derived seagrass classification maps to local seagrass coverage results (Table 1; Table S2). These datasets represent secondary data, meaning data collected by other organizations which was made publicly available and used here. When selecting reference data at each study area, priority was given to datasets with minimal temporal offsets with satellite imagery acquisition. Comparisons between reference data and satellite classifications were made with the assistance of local experts to account for seasonal differences or local influences; Griffiths et al. (2020) encouraged engagement with local coastal management agencies or experts to fill gaps in seagrass monitoring efforts. Local seagrass coverage results were available as either point or polygon data and seagrass was classified as either seagrass presence or seagrass percentage cover, where seagrass percentage cover included various density classes depending on the data source (Table S2).

Local seagrass coverage data are hereafter referred to as reference data; however, reference data are not error-free, and several limitations must be recognized when comparing reference data to our satellite image classifications. First, reference data were not always validated through on-the-ground measurements. Second, reference data used in this study were not contemporaneous with satellite overpass, meaning seasonal and annual offsets existed between local data collection and satellite image acquisition. Finally, spatial mismatches between data collection procedures complicated statistical comparisons because reference data were often collected as point or polygon shapefiles, while satellite imagery is raster-based.

### 2.3. Satellite-estimated seagrass coverage

#### 2.3.1. Satellite imagery

Satellite data were obtained from Maxar’s WorldView-2 and WorldView-3 commercial satellite platforms (Maxar, 2019) through the NASA Commercial Smallsat Data Acquisition Program’s NextView License agreement. This agreement between the National Geospatial-Intelligence Agency and Maxar makes data from select commercial satellite platforms available to the United States Federal Government, which may be shared according to the NextView License (NASA, 2022b). WorldView-2 was launched in October 2009, WorldView-3 was launched in August 2014, and both are still operational. Each satellite offers six spectral bands in the visible wavelengths and two spectral bands in the near-infrared wavelengths (Table S3). WorldView-2 data were prioritized over WorldView-3, as WorldView-2 offers a higher signal-to-noise ratio than WorldView-3, making it more appropriate for aquatic applications (Coffer et al., 2022). WorldView-2 imagery is collected at a spatial resolution of 1.84 m at nadir, where nadir is defined as the point on Earth’s surface directly below the satellite, while WorldView-3 offers a slightly improved spatial resolution of 1.24 m at nadir.

Data were obtained from Maxar’s Global Enhanced Geospatial Intelligence Delivery service (evwhs.digitalglobe.com). For each study area, a single scene was selected based on minimal cloud cover, an off- nadir view angle to avoid sun glint issues (Vanhellemont, 2019; Vanhellemont and Ruddick, 2018), and visibly ideal water clarity (Table 1, Table S2). While low tide is typically preferred for seagrass mapping, satellite data often lacks sufficient temporal coverage to select images based on tidal stage (Table S2; Table S4). Prioritization was given to satellite scenes that corresponded temporally to reference data described in Section 2.2, although exact temporal matches were limited due to their inconsistent temporal coverage. Following Coffer et al. (2020), each satellite image was processed from Level 1B data to produce an orthorectified, radiometrically corrected, and atmospherically corrected scene as remote sensing reflectances $\left(R_{\text{rs}}\right)$ in units of per steradian $\left({\text{sr}}^{-1}\right)$. For atmospheric correction, Coffer et al. (2020) used a Rayleigh exponent of 4.75. The same was done for all sites here, with three exceptions. At South Padre Island, Texas (TX), Broad Sound, Massachusetts (MA), and Nahant Bay, MA, a Rayleigh exponent of 4 was used to account for hazy conditions at the time of image acquisition (Chavez, 1988; Curcio, 1961; Slater et al., 1983). Satellite data processing was performed in Python (Python Core Team, 2015).

#### 2.3.2. Classifying satellite imagery

A deep convolutional neural network (DCNN) was used for image classification because it provides a good balance between computational complexity and model performance (Islam et al., 2018, 2020). A DCNN model essentially generates many moving windows across an image to identify features such as edges, curves, and colors. The DCNN model required some input knowledge for training, which was provided as spectral information contained within user-defined regions of interest (ROIs). ROIs were defined for every class in each satellite image independent of reference data described in Table 1. Instead, ROIs were defined based on local expert knowledge, expected spectral shape, and visual confirmation. This DCNN model was successfully used to classify seagrass using satellite imagery from WorldView-2, WorldView-3, RapidEye, and Landsat 5 through 8 (Coffer et al., 2020; Lebrasse et al., 2022), and was developed using the Keras package in Python (Chollet, 2015).

Optical properties of the water column and characteristics of the littoral zone varied across study areas. Thus, a unique collection of classes was defined for each study area, which was then collapsed into four general classes—seagrass, no seagrass, land, and no data—to allow for comparisons across study areas (Table S5). The no seagrass category represented satellite pixels in which the littoral zone was visible but did not contain seagrass. The no data category represented satellite pixels in which the water column was obfuscated, producing a detectable $R_{\text{rs}}$ signal but preventing characterization of the aquatic substrate. Satellite pixels categorized as no data did not preclude the presence of seagrass, meaning that seagrass could be present for those satellite pixels, but was not visible for the given satellite image. Some satellite images did not require a no data class because water clarity and depth made the entire aquatic substrate visible. Currently, multispectral satellite data is insufficient for differentiating seagrass from spectrally similar habitats such as algae (Kutser et al., 2020). Additionally, algal and seagrass habitats are often intermixed, further complicating their optical separation. Yet algae can be an important driver of seagrass coverage along the eutrophication gradient (Han et al., 2016). While recent research efforts have attempted to spectrally separate seagrass and algae (Kuhwald et al., 2022), reference data used here did not categorize algae across all study areas and therefore was not considered as a separate class in the image classification.

### 2.4. Comparing satellite and reference data

**Fig. 2.**
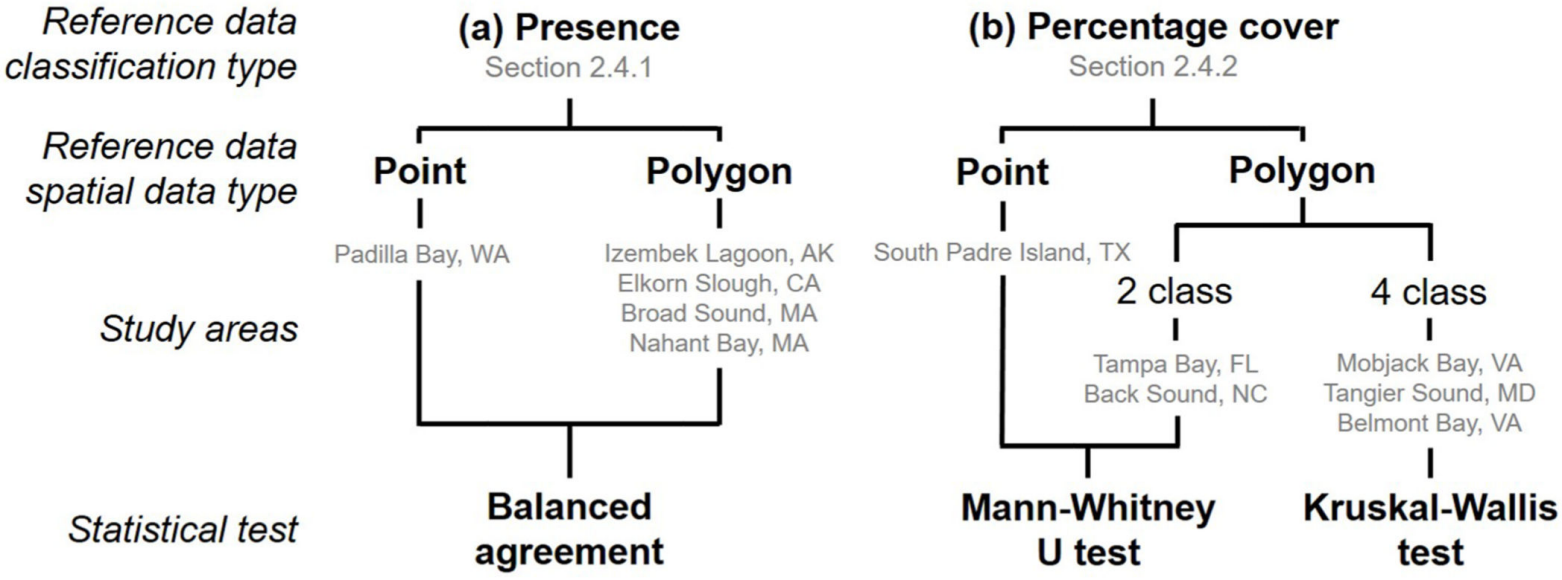
A flowchart indicating the statistical test used to assess agreement between satellite and reference data when reference data specified (a) seagrass presence and (b) seagrass percentage cover.

Agreement was assessed between satellite and reference data, although there are inherent differences in reference data collection methods and satellite image classification which can limit comparability between datasets. Depending on the study area, reference data were available as either points or polygons that specified either seagrass presence or seagrass percentage cover (Table S2). As such, agreement was quantified differently depending on the seagrass classification type and spatial data type of the reference data (Fig. 2). Satellite pixels classified as no data were excluded before comparison. Note, the term agreement is used throughout in place of accuracy as both the satellite and reference datasets being compared represent observations that have not always been validated through independent ground-based measurements.

#### 2.4.1. Reference data indicating seagrass presence

Reference data obtained for Izembek Lagoon, Alaska (AK), Padilla Bay, Washington (WA), Elkhorn Slough, California (CA), Broad Sound, MA, and Nahant Bay, MA, specified seagrass presence (Fig. 2a). Reference data were provided as point data at Padilla Bay, WA, and as polygon data at the remaining study areas. Reference data were first rasterized to match the spatial resolution of the satellite imagery. Balanced agreement was used to assess the performance of the satellite classifications, which allows for imbalanced class sizes between the binary classes being compared (Chawla et al., 2004; Daskalaki et al., 2006). Balanced agreement is computed as the arithmetic mean of sensitivity and specificity; sensitivity is the proportion of total satellite pixels classified as seagrass where both datasets indicated seagrass presence, and specificity is the proportion of total satellite pixels classified as no seagrass where both datasets indicated seagrass absence. Balanced agreement ranges from 0% to 100%, where 100% indicates complete agreement between satellite and reference data (Velez et al., 2007), and was computed using the *caret* package (Kuhn et al., 2020) in R (R Core Team, 2021).

#### 2.4.2. Reference data indicating seagrass percentage cover

Currently, there is no generally accepted method for assessing agreement between pixel-based classifications and either multi-class or continuous reference data; methods presented here offer a statistical framework for comparing such datasets. Reference data obtained for South Padre Island, TX, specified continuous seagrass percentage cover and reference data obtained for Tampa Bay, Florida (FL), Back Sound, North Carolina (NC), Mobjack Bay, Virginia (VA), Tangier Sound, Maryland (MD), and Belmont Bay, VA, specified ordinal seagrass percentage cover (Fig. 2b). Reference data at South Padre Island, TX, were provided as points spanning 0%–100% seagrass percentage cover. At this study area, the non-parametric Mann-Whitney U test was used to assess if reference-indicated seagrass percentage cover was greater in satellite pixels classified as seagrass than satellite pixels classified as no seagrass (Mann and Whitney, 1947; Wilcoxon, 1945). Results of the Mann-Whitney U test were further distilled into an effect size following the Glass (1966) formulation of rank-biserial correlation $\left(r_{\text{rb}}\right)$. The resulting effect size was then classified according to the scheme introduced by Cohen (1988) for correlation coefficients where $0.1\leq \left|r_{\text{rb}}\right|<0.3$ indicates a small association between datasets, $0.3\leq \left|r_{\text{rb}}\right|<0.5$ indicates a moderate association, and $\left|r_{\text{rb}}\right|\geq 0.5$ indicates a large association. Large associations between datasets indicate results have practical significance, while small associations indicate limited practical applications.

At Tampa Bay, FL, and Back Sound, NC, reference data were provided as polygons with two ordinal density classes: patchy and continuous (Table S6). At Tampa Bay, FL, seagrass cover between 25% and 75% was defined as ‘patchy,’ and seagrass cover between 75% and 100% was defined as ‘continuous’ (Sherwood et al., 2017). At Back Sound, NC, seagrass cover between 5% and 70% was defined as ‘patchy,’ and seagrass cover between 70% and 100% was defined as ‘continuous.’ At both of these study areas, seagrass cover less than the lower bound of the patchy seagrass class—25% at Tampa Bay, FL, and 5% at Back Sound, NC—was not delineated in the reference data. For comparison to reference data, the satellite classification was first clipped to the boundary of each individual reference polygon (Fig. S1). Reference polygons with more than 90% of their data classified as no data or with less than ten pixels remaining after excluding those categorized as no data were discarded before analysis. Next, percentage cover was computed within each individual reference polygon as the percentage of satellite pixels classified as seagrass out of all valid satellite pixels, where valid satellite pixels were those classified as either seagrass or no seagrass (*i.e.*, not classified as no data or land). This resulted in two variables for each reference polygon: an ordinal variable specifying one of two reference-indicated density classes and a continuous variable specifying satellite-indicated percentage cover. At Tampa Bay, FL, and Back Sound, NC, the non-parametric Mann-Whitney U test was used to assess if the satellite-indicated seagrass percentage cover for the continuous density class was greater than for the patchy density class. Again, $r_{\text{rb}}$ was used to further distill results of the Mann-Whitney U test into an effect size and was interpreted following Cohen (1988).

At Mobjack Bay, VA, Tangier Sound, MD, and Belmont Bay, VA, reference data were provided as polygons with four ordinal density classes: 1%–10%, 11%–40%, 41%–70%, and 71%–100% (Table S6). Again, the satellite classification was clipped to the boundary of each individual reference polygon and percentage cover was computed (Fig. S1). This resulted in two variables for each reference polygon: an ordinal variable specifying one of four reference-indicated density classes and a continuous variable specifying satellite-indicated percentage cover. At Mobjack Bay, VA, Tangier Sound, MD, and Belmont Bay, VA, the non-parametric Kruskal-Wallis test was used to assess agreement between the two datasets (Kruskal and Wallis, 1952). The Kruskal-Wallis test assessed if satellite-indicated seagrass percentage cover for density classes of 1%–10% seagrass cover, 11%–40%, 41%–70%, and 71%–100% were generated from the same population. Results of the Kruskal-Wallis test were further distilled into an effect size following the Kelley (1935) formulation of epsilon-squared $\left({\text{ε}}^{2}\right)$. The resulting effect size was classified according to a modified version of the scheme first introduced by Cohen (1988), where $0.01\leq \left|{\text{ε}}^{2}\right|<0.08$ indicates a small association between datasets, $0.08\leq \left|{\text{ε}}^{2}\right|<0.26$ indicates a moderate association, and $\left|{\text{ε}}^{2}\right|\geq 0.26$ indicates a large association (Mangiafico, 2016).

Like Analysis of Variance, the Kruskal-Wallis test only determines if there is variation in satellite-indicated seagrass percentage cover between two or more density classes. Therefore, when the Kruskal-Wallis test demonstrated that substantive variation was present, post hoc pairwise Mann-Whitney U tests were performed to sequentially compare satellite-indicated seagrass percentage cover between each of the four density classes. The Mann-Whitney U tests were two-tailed, and allowed for comparison between satellite and reference data to determine if higher estimates of satellite-indicated seagrass percentage cover were associated with the higher seagrass density classes in the reference datasets. Rank-biserial correlation was computed using the *effectsize* package (Ben-Shachar et al., 2020) and ε^2^ was computed using the *FSA* package (Ogle et al., 2001), both in R (R Core Team, 2021).

### 2.5. Instructional videos

Instructional videos detailing the processing workflow applied at each study area were generated to assist stakeholders in the transition from traditional field- and aerial-based mapping efforts to the inclusion of satellite data as a monitoring tool for seagrass ecosystems. Instructional Video 1 illustrates data selection and download using web-based data archives (Section 2.3.1). Instructional Video 2 outlines data processing to convert basic imagery to an analysis-ready product using open-source Python programming (Section 2.3.1). Instructional Video 3 details the generation of scene-specific ROIs, via ESRI’s ArcGIS Pro, and image classification, via Python (Section 2.3.2). These instructional videos, required processing scripts, and example data are available for download at DOI:10.23719/1528146.

## 3. Results

### 3.1. Seagrass classification at each study area

**Fig. 3.**
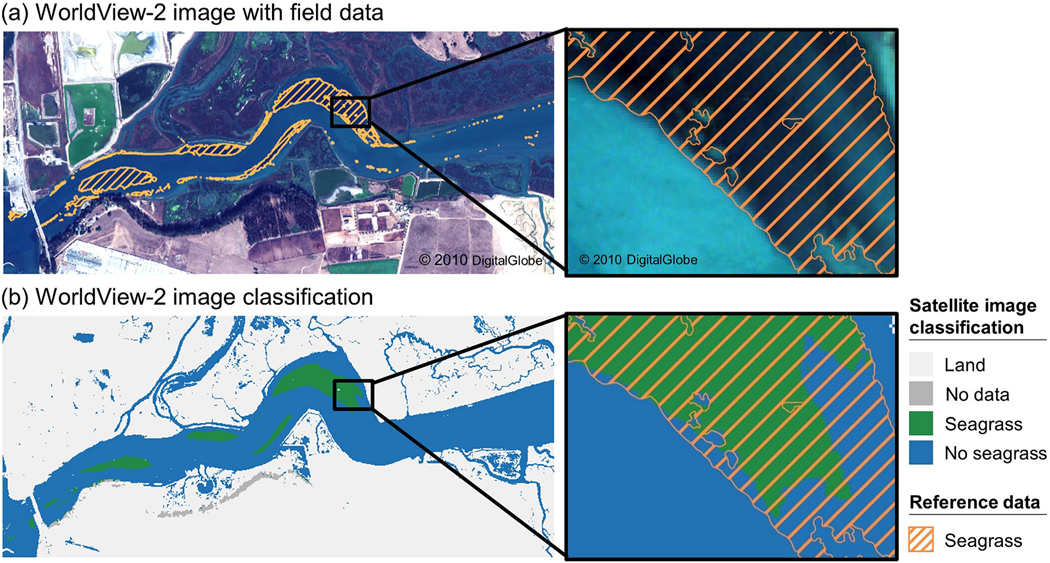
Seagrass classification results for Elkhorn Slough, California, located in the Temperate North Pacific seagrass bioregion: (a) A WorldView-2 satellite image acquired on 28 October 2017 overlaid with reference data delineating seagrass presence collected in summer 2018 and (b) image classification results for the WorldView-2 image shown in (a).

**Fig. 4.**
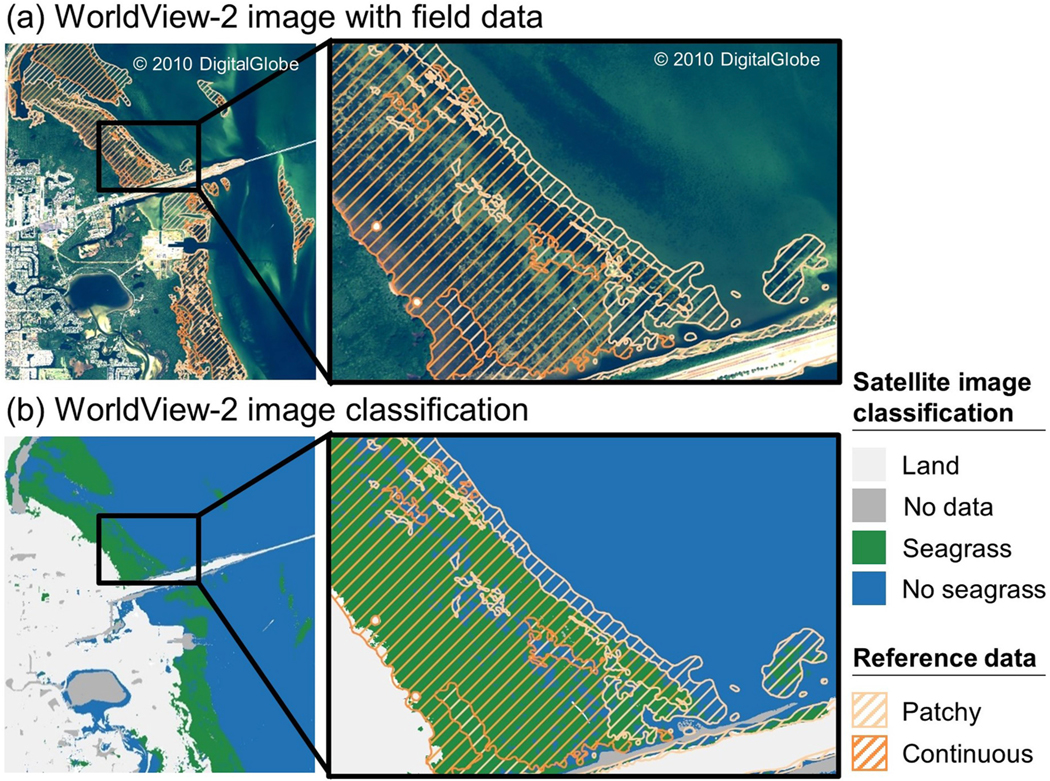
Seagrass classification results for Tampa Bay, Florida, located in the Tropical Atlantic seagrass bioregion: (a) A WorldView-2 satellite image acquired on 17 November 2016 overlaid with reference data delineating patchy and continuous seagrass collected in January 2016 and (b) image classification results for the WorldView-2 image shown in (a).

**Fig. 5.**
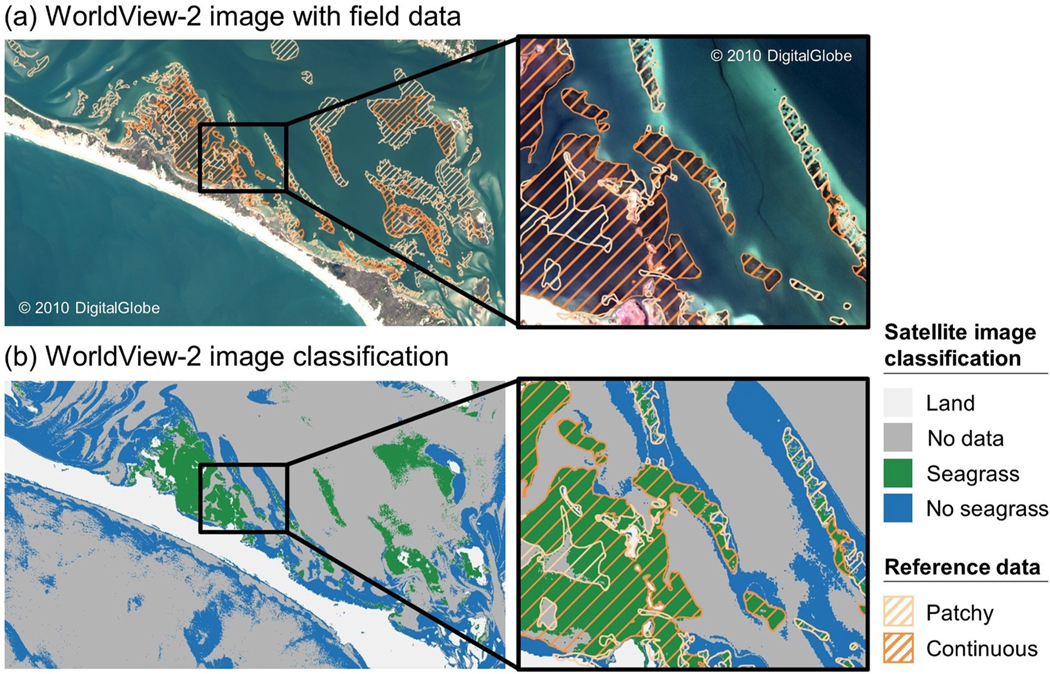
Seagrass classification results for Back Sound, North Carolina, located in the Temperate North Atlantic seagrass bioregion: (a) A WorldView-2 satellite image acquired on 27 March 2013 overlaid with reference data delineating patchy and continuous seagrass collected in May 2013 and (b) image classification results for the WorldView-2 image shown in (a).

The satellite classification performed best in dense, continuous seagrass meadows and served as a suitable spatial representation of seagrass distribution within each of the eleven study areas (Figs. 3–5; Figs. S2–S9). The satellite classification at Tampa Bay, FL, was particularly adept at capturing the boundary of seagrass beds, including differentiating relatively small patches of sand, rock, and algae from neighboring seagrass (Fig. 4). At Back Sound, NC, the satellite classification performed well in both patchy and continuous seagrass beds when water clarity and depth allowed characterization of the substrate (Fig. 5). Many satellite pixels in this image were classified as no data, preventing classification as either seagrass or no seagrass, a limitation at several other study areas as well, including Mobjack Bay, VA (Fig. S5), Tangier Sound, MD (Fig. S6), and Broad Sound, MA (Fig. S8).

Disagreement between satellite classification and reference data occurred primarily in areas of sparse, discontinuous seagrass. At Elkhorn Slough, CA, large seagrass meadows across the study area were captured by the satellite data, but smaller reference polygons along the southern shore of the Slough and in the eastern part of the satellite image were not captured (Fig. 3). Additionally, the image classification at Elkhorn Slough, CA, did not fully capture the transition between seagrass and bare sand in deeper regions. At Izembek Lagoon, AK, patchy seagrass in the northeast corner of the satellite image was not captured in its entirety (Fig. S2). Likewise, satellite-indicated seagrass was often not captured within reference-indicated low seagrass density polygons across the Chesapeake Bay study areas (Mobjack, Bay, VA, Tangier Sound, MD, and Belmont Bay, VA; Figs. S5–S7). At Tampa Bay, FL, disagreement occurred primarily along the shoreline where reference data indicated both patchy and continuous seagrass, but the satellite classification indicated land. Some instances of overclassification occurred outside of reference polygons, largely due to cloud cover. Cloud-free satellite images were preferred, but some cloud cover remained at Izembek Lagoon, AK, Tangier Sound, MD, and Nahant Bay, MA. Moreover, neither reference data nor the satellite image classifications included algae in their delineations; therefore, it is possible that areas classified as seagrass could be capturing benthic algae.

### 3.2. Agreement with reference data

#### 3.2.1. Seagrass presence agreement assessment

Balanced agreement ranged between 58% and 86% across the five study areas whose reference data indicated seagrass presence and absence (Table 2), although collection methods for reference and satellite data differed, which may limit comparability. Balanced agreement was relatively high, 81%, at Izembek Lagoon, AK, despite a large temporal disparity between datasets. Balanced agreement at Padilla Bay, WA, was 76%, although reference data were provided as point measurements and therefore do not represent the entire satellite classification area. Elkhorn Slough, CA, had the highest balanced agreement of these five study areas at 86%. Balanced agreement was lowest at the two study areas in MA; at Broad Sound balanced agreement was 72% and at Nahant Bay, 58%. Specificity was considerably higher than sensitivity across these five study areas, indicating better agreement between reference- and satellite-indicated seagrass absence than between reference- and satellite-indicated seagrass presence.

#### 3.2.2. Seagrass percentage cover agreement assessment

**Fig. 6.**
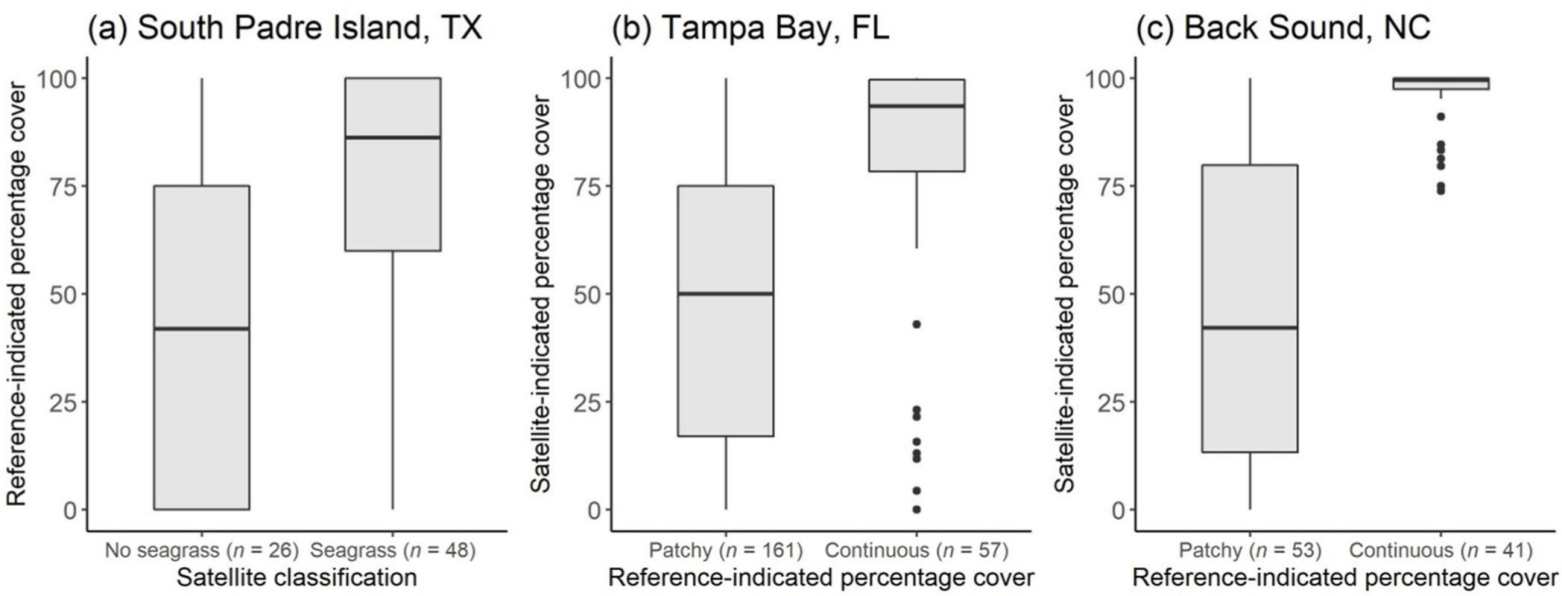
Boxplots representing (a) reference-indicated seagrass percentage cover corresponding to satellite-derived classifications of no seagrass and seagrass at South Padre Island, Texas (TX), and satellite-indicated seagrass percentage cover corresponding to reference-indicated patchy and continuous seagrass percentage cover at (b) Tampa Bay, Florida (FL), and (c) Back Sound, North Carolina (NC).

At South Padre Island, TX, results of the Mann-Whitney U test and associated rank-biserial correlation demonstrated that reference-indicated seagrass percentage cover was substantially higher in satellite pixels classified as seagrass than in satellite pixels classified as no seagrass, indicating a strong correlation between datasets (Fig. 6a; Table 3; U=291, N=74, $\left|r_{\text{rb}}\right|=0.53$). For satellite pixels classified as no seagrass, reference data had a median seagrass percentage cover of 42%. As expected, reference-indicated seagrass percentage cover corresponding to satellite pixels classified as seagrass was higher, with a median of 86%.

At Tampa Bay, FL, results of the Mann-Whitney U test and associated rank biserial correlation demonstrated that satellite-indicated seagrass percentage cover was substantially higher in reference-indicated continuous seagrass polygons than in reference-indicated patchy seagrass polygons, indicating a strong correlation between datasets (Fig. 6b; Table 3; U=2204.5, N=218, $\left|r_{\text{rb}}\right|=0.52$). Satellite-indicated seagrass percentage cover within patchy seagrass polygons had a median of 50%, equivalent to the average of the reference-indicated patchy seagrass class upper and lower bounds, which spans 25%–75%. Satellite- indicated seagrass percentage cover within continuous seagrass polygons had a median of 94%, within the reference data range of 75%–100%. However, several statistical outliers exist where satellite-indicated seagrass percentage cover corresponding to reference-indicated continuous seagrass was below 50%.

Similarly, at Back Sound, NC, results of the Mann-Whitney U test and associated rank biserial correlation demonstrated that satellite-indicated seagrass percentage cover was substantially higher in reference-indicated continuous seagrass polygons than in reference- indicated patchy seagrass polygons, indicating a strong correlation between datasets (Fig. 6c; Table 3; U=189, N=74, $\left|r_{\text{rb}}\right|=0.83$). Satellite- indicated seagrass percentage cover within patchy seagrass polygons had a median of 42%, consistent with the reference data range of 5%–70%. Satellite-indicated seagrass percentage cover within continuous seagrass polygons had a median of 100%. Statistical outliers in satellite- indicated seagrass percentage cover existed below approximately 95%, but all statistical outliers were still within the reference data range of 70%–100%.

After removing reference polygons with more than 90% of their data classified as no data and those with less than 10 valid satellite pixels, insufficient data remained at Belmont Bay, VA, for statistical analysis. For reference-indicated percentage cover classes of 1%–10%, 11%–40%, 41%–70%, and 71%–100%, sample sizes were 1, 2, 1, and 16, respectively. The minimum sample size suggested for applying the Kruskal- Wallis test to compare four classes is 24 (Dwivedi et al., 2017), with a minimum of 5 observations per group (Mundry and Fischer, 1998). Since these sample size criteria were not met, the classification at Belmont Bay, VA, was not statistically assessed using the Kruskal-Wallis test, but average satellite-indicated seagrass percentage cover was compared qualitatively to reference data. At Belmont Bay, VA, satellite imagery slightly underestimated seagrass percentage cover when compared to reference data for the lowest three density classes. Satellite-indicated seagrass percentage cover was 0% for the one reference-indicated polygon with 1%–10% seagrass cover, had a median of 3% for the two reference-indicated polygons with 11%–40% seagrass cover, and was 21% for the one reference-indicated polygon with 41%–70% seagrass cover. For the highest density class, satellite-indicated seagrass percentage cover was within the reference data range, with a median of 72%.

**Fig. 7.**
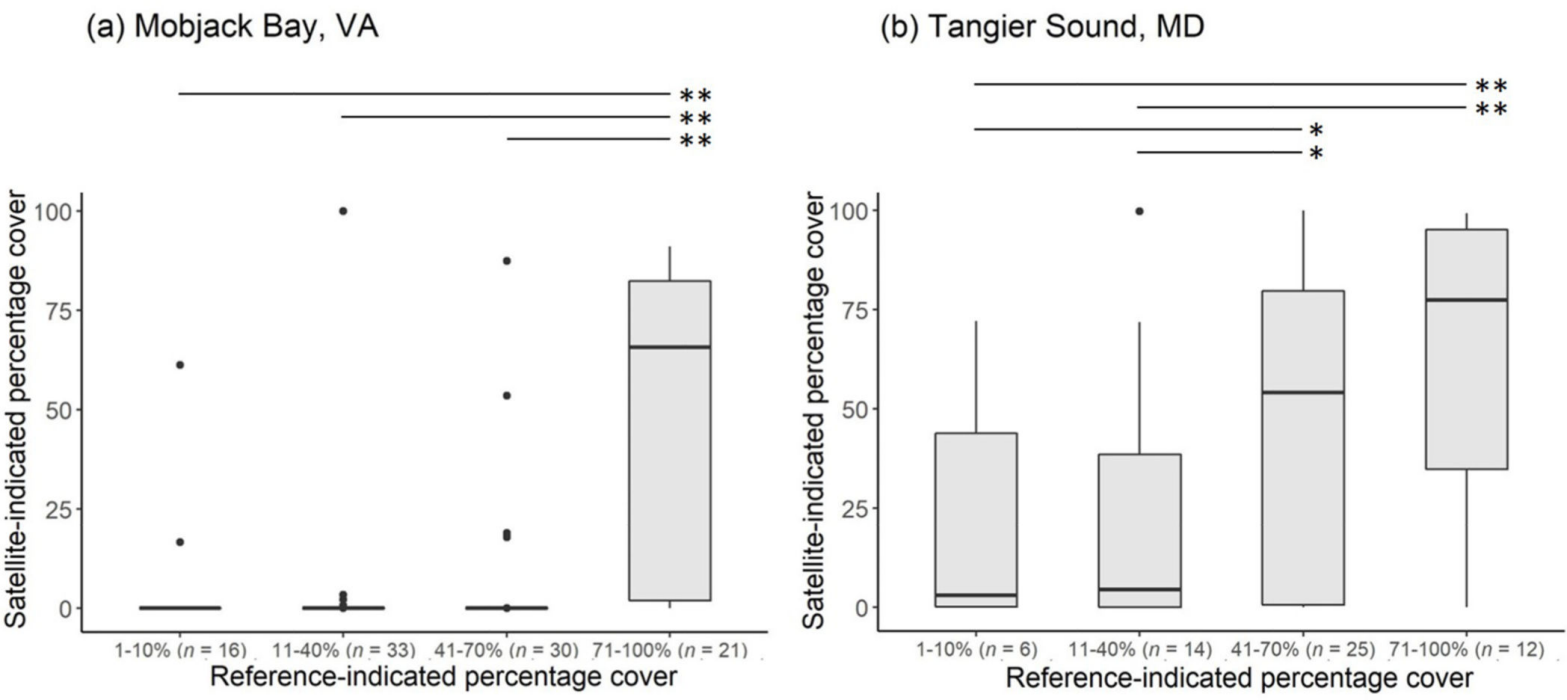
Boxplots representing satellite-indicated seagrass percentage cover corresponding to reference-indicated seagrass percentage cover classes at (a) Mobjack Bay, Virginia (VA), and (b) Tangier Sound, Maryland (MD). Horizontal lines above each set of boxplots illustrate results of the post hoc pairwise Mann-Whitney U test, where moderate (*) and large (**) associations between classes were found (see Table S7).

At Mobjack Bay, VA, results of the Kruskal-Wallis test and associated epsilon-squared demonstrated that associations between satellite-indicated seagrass percentage cover across the four seagrass density classes were large (Fig. 7a; Table 3; $\chi^{2}=31.74$, N=100, $\left|\varepsilon^{2}\right|=0.14$). Since associations were large, the post hoc pairwise Mann-Whitney U test was applied (Fig 7a; Table S7). Results showed that satellite- indicated seagrass percentage cover was higher within reference polygons in the highest seagrass density class (71%–100%) than reference polygons with 1%–10% (U=53, N=37, $\left|r_{\text{rb}}\right|=0.68$), 11%–40% (U=119, N=64, $\left|r_{\text{rb}}\right|=0.66$), and 41%–70% (U=114, N=51,$\left|r_{\text{rb}}\right|=0.64$) seagrass cover. Satellite imagery underestimated seagrass percentage cover compared to reference data within the lowest three density classes; all satellite-indicated seagrass percentage covers had a median of 0%, although statistical outliers at higher seagrass percentage covers existed. For the reference-indicated highest density class, satellite- indicated seagrass percentage cover had a median of 66%, just below the reference data range of 71%–100%.

At Tangier Sound, MD, associations between satellite-indicated seagrass percentage cover across the four seagrass density classes were moderate (Fig. 7b; Table 3; $\chi^{2}=7.59$, N=57, $\left|\varepsilon^{2}\right|=0.32$). The post hoc pairwise Mann-Whitney U test showed that satellite-indicated seagrass percentage cover was substantially higher within reference polygons in the highest seagrass density class (71%–100%) than reference polygons with 1%–10% (U=16, N=18, $\left|r_{\text{rb}}\right|=0.56$) and 11%–40% (U=40, N=26, $\left|r_{\text{rb}}\right|=0.52$) seagrass cover (Fig. 7b; Table S7). Substantial associations were also found between the third density class (41%–70%) and both 1%–10% (U=49, N=31, $\left|r_{\text{rb}}\right|=0.35$) and 11%–40% (U=110, N=39, $\left|r_{\text{rb}}\right|=0.37$) seagrass cover, although these associations were moderate. Satellite-indicated seagrass percentage cover within reference polygons with 1%–10% cover and 11%–40% cover were similar, with medians of 3% and 5%, respectively, and both fell within the reference data range for the lowest density class. For reference polygons with 41%–70% seagrass cover, satellite results had a median of 54% and for those with 71%–100% seagrass cover, a median of 78%, both of which were within the reference data ranges.

## 4. Discussion

Satellite imagery from WorldView-2 and WorldView-3 was used to classify seagrass presence and absence at eleven coastal study areas across the continental United States, advancing satellite remote sensing of seagrass ecosystems to NASA’s second validation stage, which describes when a product’s performance has been assessed against field programs over additional locations and time periods (NASA, 2022a). This study presents a spatially comprehensive assessment of satellite remote sensing for seagrass mapping, marking a significant step toward the use of satellite imagery to refine global estimates of seagrass coverage (Duarte, 2017) and carbon storage capacity (Fourqurean et al., 2012), and to manage and protect seagrasses and their ecosystem services (Bell et al., 2007; Duarte, 2002; Neckles et al., 2012). This study assessed agreement between satellite image classifications and spatially concurrent reference data; understanding differences between these datasets is critical for improving future assessments of satellite classification performance and for understanding appropriate applications of satellite imagery as a complementary management tool for monitoring seagrass ecosystems.

Satellite image classification performed best in areas of continuous seagrass. Balanced agreement indicated that similarities between satellite classifications and reference datasets ranged from 58% to 86%, and seagrass absence was classified with better agreement than seagrass presence. Comparisons at Izembek Lagoon, AK, and Belmont Bay, VA, are particularly suitable given reference data at each of these study areas was generated using satellite remote sensing. Seagrass presence at Izembek Lagoon, AK, was delineated from 30 m Landsat imagery (Hogrefe et al., 2014). Seagrass density ranges at Belmont Bay, VA, were photointerpreted from the same WorldView-2 scene used in this study (Orth et al., 2019), although insufficient sample size prevented a statistical comparison of results at this study area.

Disagreement between satellite image classifications and reference data in areas of sparse seagrass can be the result of overestimation of seagrass coverage in reference datasets (Meehan et al., 2005) or underestimation of seagrass coverage through image classification. Additional data would be needed to quantify each of these components. At study areas whose reference data indicated seagrass presence and absence, relatively low sensitivity, ranging from 17% to 73%, compared to specificity, ranging from 88% to 100%, suggested heterogeneity within seagrass reference polygons. The Mann-Whitney U and Kruskal-Wallis tests demonstrated that satellite data can be used to map seagrass across varying coverage densities, although performance was best in areas with high seagrass percentage cover. Underestimation of seagrass cover at Izembek Lagoon, AK, occurred in areas with less than approximately 30% seagrass cover (Ward and Amundson, 2019).

Patchy seagrass can be an important indicator of either increasing (Bell et al., 2007) or decreasing seagrass coverage (Duarte et al., 2007), but patchy seagrass is difficult to characterize with both satellite remote sensing (Baumstark et al., 2016; Carpenter et al., 2022; Green et al., 1996; Hill et al., 2014; Knudby and Nordlund, 2011; Phinn et al., 2008; Pu et al., 2014; Pu and Bell, 2013) and aerial imagery (Kaufman and Bell, 2020; McKenzie et al., 2001; Meehan et al., 2005). In satellite imagery, patchy seagrass is likely underestimated because each satellite pixel captures the average brightness for all features within that pixel (Bhatta, 2013). Even though many satellite pixels can contain a mix of several classes, especially in patchy seagrass environments, the DCNN used in this study assigns one class to each pixel. In some cases, smaller features can dominate the brightness within a satellite pixel, becoming detectable even at sub-pixel scales (Brown et al., 2000; Phinn et al., 2002; Xian and Crane, 2005). Therefore, satellite pixels classified as no seagrass do not necessarily represent 0% seagrass coverage, but instead indicate that the spectral signature of other constituents within the satellite pixel outweighed the spectral signature of seagrass. Previous studies have classified patchy and continuous seagrass separately, but with lower efficacy (Knudby and Nordlund, 2011; Pu et al., 2014). The threshold at which seagrass dominates measured reflectance for a satellite pixel is not quantified (Thomas and Allcock, 1984) and can be dependent on many factors, including contrast with the surrounding environment (Barrell et al., 2015). As satellite technology continues to improve, platforms offering hyperspectral data collection in combination with high spatial resolution will likely improve classification of patchy seagrass environments.

Temporal offsets between reference and satellite data also varied widely across study areas, ranging from several months to 16 years. In the absence of temporally coincident reference and satellite data, the effect of seasonal and annual offsets between datasets cannot be quantified and interim conditions can vary widely; for example, Johnson et al. (2021) noted a substantial decline in submerged aquatic vegetation across the lower Chesapeake Bay following record rainfall and warmer temperatures in 2018. At Tampa Bay, FL, reference data was derived from aerial photography acquired in January 2016 while satellite imagery was acquired in November 2016. According to TBEP (2020), Tampa Bay seagrass coverage declined 3% from 2016 to 2018; Old Tampa Bay, the primary focus of our image classification, made up 31% of this decline. This documented decline could explain the instances of underestimation of seagrass cover compared to reference data, given the temporal offset between datasets. At Broad Sound, MA, and Nahant Bay, MA, satellite images were acquired five years after reference data was collected, and spatial shifts in seagrass cover over this time period cannot be differentiated from satellite classification error. Additionally, the satellite platform used may have decreased balanced agreement at Broad Sound, MA, and Nahant Bay, MA, as these study areas were the only two at which a WorldView-3 image was used. Coffer et al. (2022) noted that WorldView-3 could be less appropriate for aquatic applications than WorldView-2 because of its lower signal-to-noise ratio, which can decrease classification performance (Gowdra et al., 2020).

Agreement between satellite and reference data was lower in deeper areas and in instances of poor water clarity. Classification accuracy typically decreases at depth due to light attenuation (Dekker et al., 2005; Hogrefe et al., 2014; Phinn et al., 2008), which makes bottom features appear spectrally similar (Sagawa et al., 2008). Dekker et al. (2005) found that seagrass was detectable with Landsat up to depths of 2.4 m in clear water but only 0.7 m in turbid water. Kuhwald et al. (2022) found poorer classification accuracy at depths greater than 4 m. Poor water clarity prevented image-wide characterization of the aquatic substrate at several study areas, particularly those located in the Temperate North Atlantic seagrass bioregion. The no data flag proposed here acts as a quality flag to identify satellite pixels unusable for seagrass detection in each image. The ability to spectrally differentiate obfuscated water from seagrass allows satellite images to still be used even if they are collected when optical conditions within the water column are not ideal across some portion of those images. This is analogous to automated cloud masking, which removes portions of an image that are contaminated by clouds and retains the remainder of the image for subsequent analysis. In future efforts, repeated satellite image acquisitions over the same location could be mosaicked to generate a full-scene classification, exploiting more suitable optical conditions across time.

This study assessed the performance of a satellite classification approach by comparing results to reference data. However, there are important distinctions between these collection methods that may limit their comparability, primarily due to differences in spatial data types: satellite classifications are generated on a pixel-by-pixel basis, while reference data are often provided as point, line, or polygon shapefiles. Kenworthy et al. (1982) described the hypothetical gradient of seagrass bed development, which included unvegetated areas, patches of seagrass, the edge of a continuous meadow, and the interior zone of the continuous cover of vegetation. Polygon-based methods of seagrass delineation can lead to overestimation of seagrass coverage along this gradient, as areas of both seagrass and bare sand are amalgamated (Meehan et al., 2005). MassDEP, for example, generates seagrass polygons encompassing heterogeneous regions characterized by patchy seagrass intermixed with bare sand (Costello and Kenworthy, 2011); this led to low sensitivity at Broad Sound, MA, and Nahant Bay, MA, despite high specificity. VIMS classifies seagrass bed density into one of four density classes through visual comparison with a density scale that is analogous to those used for estimating forest crown cover within aerial photography (Paine and Kiser, 2012; VIMS, 2022). For study areas located in the Chesapeake Bay, the number of seagrass density classes defined in the reference data likely reduced agreement with satellite classifications. Pu et al. (2012) found worse classification performance when satellite results were parsed into 5 seagrass density classes versus 3 seagrass density classes.

The establishment of a consistent seagrass monitoring approach is crucial for closing spatial gaps in global seagrass mapping (Duffy et al., 2019; McKenzie et al., 2020). In addition to differences in data collection methods, reference data also included multiple spatial data types and seagrass classification types. Moreover, reference programs vary in their definitions of seagrass presence and bed edge. The Washington State Department of Natural Resources (WADNR) detailed existing criteria implemented by nine different agencies (WADNR, 2013). Two examples include the OSPAR Commission, which considers seagrass patches less than 10 m apart to be the same meadow, and the Tampa Bay Estuary Program, which proposed seagrass beds be defined as at least 10% cover within a 10- to 30-m transect line. Alternative definitions have also been suggested that consider seagrass seed dispersal distance, rhizome extension distance, and both historic and potential seagrass habitat. WADNR proposed seagrass mapping criteria that extends the edge of a seagrass bed 0.5 m beyond the last shoot found (WADNR, 2013). The image processing and classification regime presented here successfully classified seagrass presence and absence using a standardized, consistent approach across varying seagrass bioregions, atmospheric conditions, and optical water types, suggesting the potential for regional, and even global, applicability. In future comparisons between satellite- and reference-based seagrass maps, satellite image classifications can be revised to extend assumptions used in reference data delineation for a given area to reduce methodological differences between datasets.

Limitations associated with the application of satellite remote sensing for seagrass mapping include the inability to collect optical satellite imagery of Earth’s surface during cloudy conditions, depth constraints at which the satellite signal becomes fully attenuated, and difficulty aligning satellite image acquisition with the preferred tidal stage for seagrass mapping. Satellite overpasses typically occur around midday local time, which does not always coincide with low tide (see Table S2). This is in contrast with more opportunistic approaches like aerial image acquisition, which usually occurs within 60 to 90 minutes of low tide (Costello and Kenworthy, 2011; Orth et al., 2010). While tidal stage may not affect satellite-derived seagrass extent (Lebrasse et al., 2022), the deep edge of seagrass coverage was commonly mischaracterized in the present study due to spectral similarity at increasing water depth. Moreover, Stekoll et al. (2006) suggested biomass characterization may require image acquisition at consistent tidal stages. Finally, WorldView-2 and WorldView-3 do not continuously collect imagery due to limitations in data storage and transmission. Instead, imagery can either be acquired through their archive or by tasking the satellite for a given date and location (Coffer et al., 2022), although tasking does not guarantee a given request will be fulfilled (Landry et al., 2021). This study focused on identifying seagrass coverage but did not offer species differentiation within or among study areas. Species differentiation can be important in refining carbon storage estimates because seagrass species sequester carbon at different rates. Multispectral satellite imagery may be insufficient for achieving species identification within seagrass communities due to similar spectral characteristics. Instead, hyperspectral imagery is often needed for separating plant pigment types.

Future work should focus on expanding this demonstration across time and space. Across time, this approach can be applied to time series analyses and for regional seagrass mapping combining several overlapping satellite images. Across space, while three of six global seagrass bioregions were represented in this study, additional assessments of classification performance are necessary, particularly outside of the United States. Methods can then be extended to estimate large-scale seagrass coverage, including generating global datasets of seagrass ecosystems. Moreover, testing the transferability of ROIs could further increase accessibility to satellite imagery by reducing user input in the overall processing chain. Subsequent studies assessing satellite classification performance may consider applying a threshold to minimize the temporal offsets between reference and satellite data as interim conditions can vary widely. Additionally, as new satellite resources become available, methods presented in this study can be applied to satellite platforms offering improved resolutions. For example, Planet’s SuperDove satellite constellation can be used for more consistent data collection and offers similar spectral bands compared to WorldView-2 and WorldView-3. Future work should also include developing monitoring applications leveraging emerging satellite technology to allow for consistent, repeated images that can inform event-based, seasonal, and interannual assessments. Transferring methods presented here to satellite platforms offering more consistent and frequent image acquisition represents the next step for furthering scientific advancements and operationalizing management applications.

## 5. Conclusions

This study tested an image classification framework for seagrass presence and absence at eleven coastal study areas across the continental United States. The intent of this study was not to further validate DCNN model performance, but to demonstrate the ability to apply the same methods across multiple study areas and satellite images, representing a continental-scale range of seagrass bioregions, atmospheric conditions, and water optical properties. This marks a significant step toward developing an operational approach for mapping seagrass coverage at the national and global scales and contributes toward Stage 2 Validation as defined by NASA’s data maturity levels. The accompanying instructional videos, required processing scripts, and example data are available for download at DOI:10.23719/1528146.

Satellite imagery has the potential to complement traditional sea-grass mapping efforts but does not have the ability to fully replace localized seagrass mapping, particularly when using satellite platforms that have been evaluated in this study given their limited temporal resolution. Instead, this transition should parallel that of weather forecasting. Weather forecasting skill has increased globally over the past 40 years, primarily due to the inclusion of satellite imagery (Bauer et al., 2015). Satellite imagery and local observations are used to initiate numerical weather prediction models. The National Weather Service then generates local forecasts by analyzing and scrutinizing model output using localized, individual scientific expertise (NWS, 2022). This study offers a similar approach where satellite image classifications can be used as a standardized, initial indication of seagrass presence and absence. Local management can then adjust these pixel-based maps using their expert knowledge of specific study areas. In areas lacking localized expertise, satellite imagery can serve as an adequate representation of seagrass presence and absence to improve regional and global estimates of seagrass coverage and Blue Carbon storage (CEC, 2016). Moving forward, satellite estimates of seagrass coverage can be used in combination with known drivers of seagrass coverage such as water temperature and clarity as inputs for predictive models forecasting future changes in seagrass extent.

The information presented in this manuscript and the instructional videos included in the supplemental material can assist stakeholders in developing local seagrass monitoring programs. As general guidance, the following steps are recommended.

1. *Access Maxar imagery*: WorldView-2 or WorldView-3 imagery can be acquired either using archived imagery (Supplemental Video 1, Section 2.3.1) or by tasking the satellite for a specific date and location (Coffer et al., 2022; Landry et al., 2021).
2. *Process satellite imagery:* To increase spectral separability of classes, satellite imagery should be processed to create a radiometrically and atmospherically corrected product (Supplemental Video 2, Section 2.3.1).
3. *Classify satellite imagery:* A previously validated machine learning classifier, such as that presented in Islam et al. (2020, 2018), can be applied to the imagery to separate satellite pixels into the desired classes (Supplemental Video 3, Section 2.3.2). Classes can be tailored to fit the specifications of a location or region.
4. *Assess agreement:* This manuscript presented a statistical framework for comparing satellite and reference data (Section 2.4). Methods for both point- and polygon-based reference data of seagrass presence and percentage cover are presented and can be used to assess agreement between the machine learning classifier and other seagrass mapping methods.

## Supplementary Material

SI

## Figures and Tables

**Table 1:** Details of seagrass reference data and satellite imagery acquired at each study area. Temporal offset represents the temporal difference between reference data collection and satellite imagery acquisition, where a positive offset represents satellite imagery collected after reference data. See also Table S2.

Study area	Reference data source	Reference data collection period	Satellite imagery source	Satellite imagery acquisition date	Temporal offset
Izembek Lagoon, AK	CEC^a^	2002 to 2006	WorldView-2	12 Sept 2018	+12 to 16 years
Padilla Bay, WA	WADNR^b^	June and July 2017	WorldView-2	28 Oct 2017	+3 to 4 months
Elkhorn Slough, CA	ESF^c^ and Elkhorn Slough National Estuarine Research Reserve	Summer 2018	WorldView-2	25 Oct 2018	+2 to 5 months
South Padre Island, TX	Texas seagrass monitoring program	Late summer to Sept 2012	WorldView-2	1 Aug 2012	±0 to 1 month
Tampa Bay, FL	SFWMD^d^	January 2016	WorldView-2	17 Nov 2016	+10 months
Back Sound, NC	NCDEQ^e^	May 2013	WorldView-2	27 Mar 2013	−2 months
Mobjack Bay, VA	VIMS^f^	May to Nov 2015	WorldView-2	4 May 2015	−0 to 6 months
Tangier Sound, MD	VIMS	May to Nov 2017	WorldView-2	25 Sept 2017	±0 to 4 months
Belmont Bay, VA	VIMS	Summer 2019	WorldView-2	29 Sept 2019	+1 to 4
Broad Sound, MA	MassDEP^g^	2016	WorldView-3	7 June 2021	months +5 years
Nahant Bay, MA	MassDEP	2016	WorldView-3	7 June 2021	+5 years

a Commission for Environmental Cooperation; data from Hogrefe et al. (2014).

b Washington State Department of Natural Resources.

c Elkhorn Slough Foundation.

d Southwest Florida Water Management District.

e North Carolina Department of Environmental Quality.

f Virginia Institute of Marine Science.

g Massachusetts Department of Environmental Protection.

**Table 2:** Balanced agreement for study areas delineating seagrass presence and absence; *n* is sample size.

Study area	Reference class	Reference *n*	Sensitivity	Specificity	Agreement
Izembek Lagoon, AK	Seagrass No seagrass	17,188,571 45,228,056	67%	95%	81%
Padilla Bay, WA	Seagrass No seagrass	8244 28,911	64%	88%	76%
Elkhorn Slough, CA	Seagrass No seagrass	32,245 15,615,478	73%	100%	86%
Broad Sound, MA	Seagrass No seagrass	328,005 750,007	48%	97%	72%
Nahant Bay, MA	Seagrass No seagrass	56,069 290,421	17%	98%	58%

**Table 3:** Results of agreement assessments between satellite-derived seagrass classifications and reference-indicated seagrass; *n* is sample size. Statistical comparison was not performed at Belmont Bay, Virginia (VA), due to insufficient sample size.

Mann-Whitney U test and associated rank-biserial correlation $\left(r_{\text{rb}}\right)$					

Study area	Class	*n*	Median	$\left|r_{\text{rb}}\right|$	Association^a^
South Padre Island, TX	No seagrass	26	42%	0.53	Large
	Seagrass	48	86%		
Tampa Bay, FL	Patchy	161	50%	0.52	Large
	Continuous	57	94%		
Back Sound, NC	Patchy	53	42%	0.83	Large
	Continuous	41	100%		
		
Kruskal-Wallis test and associated epsilon-squared $\left(\varepsilon^{2}\right)$					

Study area	Class	*n*	Median	$\left|{\text{ε}}^{2}\right|$	Association^b^
Mobjack Bay, VA	1%–10%	16	0%	0.32	Large
	11%–40%	33	0%		
	41%–70%	30	0%		
	71%–100%	21	66%		
Tangier Sound, MD	1%–10%	6	3%	0.14	Moderate
	11%–40%	14	5%		
	41%–70%	25	54%		
	71%–100%	12	78%		
Belmont Bay, VA	1%–10%	1	0%	*N/A*	*N/A*
	11%–40%	2	3%		
	41%–70%	1	21%		
	71%–100%	16	72%		

a $r_{\text{rb}}$ was interpreted following Cohen (1988).

b ${\text{ε}}^{2}$ was interpreted following a variation on Cohen (1988) described in Mangiafico (2016).

## Data Availability

The accompanying instructional videos, required processing scripts, and example data are available for download at DOI:10.23719/1528146.
