# Supplementary material for: Providing a framework for seagrass mapping in United States coastal ecosystems using high spatial resolution satellite imagery
Source: J Environ Manage. Author manuscript; Available in PMC 2024 Jul 1. (PMC10622156; doi:10.1016/j.jenvman.2023.117669)
Supplement: SI [file NIHMS1888128-supplement-SI.docx]

**Supplementary Material**

**Providing a framework for seagrass mapping in United States coastal ecosystems using high spatial resolution satellite imagery**

Megan M. Coffer ^a,^ *, David D. Graybill ^a^, Peter J. Whitman ^a^, Blake A. Schaeffer ^b^, Wilson B. Salls ^b^, Richard C. Zimmerman ^c^, Victoria Hill ^c^, Marie Cindy Lebrasse ^a, d^, Jiang Li ^e^, Darryl J. Keith ^f^, Jim Kaldy ^g^, Phil Colarusso ^h^, Gary Raulerson ^i^,

David Ward ^j^, and W. Judson Kenworthy ^k^

^a^ Oak Ridge Institute for Science and Education, U.S. Environmental Protection Agency, Office of Research and Development, Durham, NC, USA

^b^ U.S. Environmental Protection Agency, Office of Research and Development, Durham, NC, USA

^c^ Department of Earth & Ocean Sciences, Old Dominion University, Norfolk, VA, USA

^d^ Department of Marine, Earth and Atmospheric Sciences, North Carolina State University, Raleigh, NC, USA

^e^ Department of Electrical and Computer Engineering, Old Dominion University, Norfolk, VA, USA

^f^ U.S. Environmental Protection Agency, Office of Research and Development, Narragansett, RI, USA

^g^ U.S. Environmental Protection Agency, Office of Research and Development, South Beach, OR, USA

^h^ U.S. Environmental Protection Agency, Office of Research and Development, Boston, MA, USA

^i^ Largo, FL, USA

^j^ U.S. Geological Survey, Alaska Science Center, Anchorage, AK, USA

^k^ Department of Biology and Marine Biology, University of North Carolina, Wilmington, NC, USA

*Corresponding author, coffermm@gmail.com

*Disclaimer: DigitalGlobe imagery was made available through the U.S. Government NextView license. Any mention of trade, firm, manufacturers, or product names does not imply an endorsement or recommendation for use by the United States Government or the U.S. Environmental Protection Agency. The views expressed in this article are those of the authors and do not necessarily reflect the views or policies of the U.S. EPA.*

**Text S1: Study area details**

Izembek Lagoon, Alaska (AK), Padilla Bay, Washington (WA), and Elkhorn Slough, California (CA), are in the Temperate North Pacific seagrass bioregion. Izembek Lagoon, AK, is located on the Bering Sea side of the Alaska Peninsula in the Izembek National Wildlife Refuge. Water clarity at Izembek Lagoon, AK, is favorable for optical measurements, with Secchi depths averaging 4 m (Hogrefe et al., 2014). However, frequent cloud cover limits airborne or spaceborne data collection, with only 12 clear days per year on average (Taylor and Sowl, 2007). Padilla Bay, WA, is an estuary of Puget Sound. While water clarity in Puget Sound increased from 2008 through 2018 (PSEMP Marine Waters Workgroup, 2020), waters remain impaired (Puget Sound Partnership, 2019). Elkhorn Slough, CA, is a tidal slough and estuary that opens to Monterey Bay. Seagrasses at Elkhorn Slough, CA, can be difficult to observe at depth due to high turbidity and strong tidal currents (Dierssen et al., 2019).

South Padre Island, Texas (TX), and Tampa Bay, Florida (FL), are in the Tropical Atlantic seagrass bioregion. South Padre Island, TX, is a barrier island at the southern end of Lower Laguna Madre. Despite increased nutrient pollution throughout Lower Laguna Madre (Kuwayama et al., 2020), water depths in the lagoon are shallow, averaging only 1 m (Tunnell Jr., 2002), making the littoral zone relatively easy to observe using optical methods. Tampa Bay, FL, opens to the Gulf of Mexico. Waters in Tampa Bay, FL, can be optically challenging given the high levels of colored dissolved organic matter (CDOM) derived from terrestrial and wetland vegetation (Erickson et al., 2004), and nutrient-fueled phytoplankton blooms (McPherson and Miller, 1994).

Back Sound, North Carolina (NC), Mobjack Bay, Virginia (VA), Tangier Sound, Maryland (MD), Belmont Bay, VA, Broad Sound, Massachusetts (MA), and Nahant Bay, MA, are in the Temperate North Atlantic seagrass bioregion. Although defined as Temperate North Atlantic, the estuary at Back Sound, NC, represents a unique transition between temperate and subtropical zones with both temperate and tropical seagrass species dominating, depending on the time of year. Thus, while Back Sound, NC, is included in the Temperate North Atlantic seagrass bioregion, it differs ecologically from the remaining study areas included in this bioregion. Waters in Back Sound, NC, are well-mixed and relatively shallow, averaging 2 m in depth (Bartenfelder et al., 2022). Mobjack Bay, VA, Tangier Sound, MD, and Belmont Bay, VA, are located in Chesapeake Bay, the largest estuary in the United States. Water clarity throughout Chesapeake Bay varies spatially and seasonally, but waters are typically turbid (Turner et al., 2021), and water transparency has decreased since the 1980’s (Testa et al., 2019). On average, Mobjack Bay, VA, near the mouth of Chesapeake Bay, has improved water clarity compared to Tangier Sound, MD, and Belmont Bay, VA, which are closer to the Bay’s eutrophic headwaters (Testa et al., 2019). Poor water clarity in combination with high-amplitude, wind-driven tidal fluctuations (Xiong and Berger, 2010) can make optical detection of the littoral zone in Chesapeake Bay challenging. Broad Sound, MA, lies just north of the city of Boston and is separated by a peninsula from Nahant Bay, MA, to the northeast. While Nahant Bay, MA, is characterized by clear, well-flushed waters, Broad Sound, MA, has higher levels of CDOM due to influx from the Pines and Saugus Rivers.

Table S1. Characteristics of each study site, including seagrass bioregion as defined by Short et al. (2007), climate region as defined by Karl and Koss (1984), and dominant seagrass species.

| **Study area** | **Seagrass bioregion** | **Climate region** | **Dominant seagrass species** | **Citation** |
| --- | --- | --- | --- | --- |
| Izembek Lagoon, AK | Temperate North Pacific | *N/A* | *Zostera marina* | (Ward et al., 1997) |
| Padilla Bay, WA | Temperate North Pacific | Northwest | *Zostera marina* | (Christiaen et al., 2019) |
| Elkhorn Slough, CA | Temperate North Pacific | West | *Zostera marina* | (Hammerstrom and Grant, 2012) |
| South Padre Island, TX | Tropical Atlantic | South | *Thalassia testudinum* | (Onuf, 2007) |
| Tampa Bay, FL | Tropical Atlantic | Southeast | *Halodule wrightii* | (Sherwood et al., 2017) |
| Back Sound, NC | Temperate North Atlantic | Southeast | *Zostera marina* (summer); *Halodule wrightii* (winter) | (Bartenfelder et al., 2022) |
| Mobjack Bay, VA | Temperate North Atlantic | Southeast | *Zostera marina* | (Orth et al., 2010) |
| Tangier Sound, MD | Temperate North Atlantic | Southeast | *Zostera marina* | (Orth et al., 2010) |
| Belmont Bay, VA | Temperate North Atlantic | Southeast | *Zostera marina* | (Orth et al., 2010) |
| Broad Sound, MA | Temperate North Atlantic | Northeast | *Zostera marina* | (Carman et al., 2019) |
| Nahant Bay, MA | Temperate North Atlantic | Northeast | *Zostera marina* | (Carman et al., 2019) |

Table S2. Additional details of seagrass refence data and satellite imagery acquired at each study area. Acquisition time represents the local time of satellite image acquisition. Tidal height represents tidal predictions acquired from the nearest station (Table S4) through the NOAA Tides & Currents database (https://tidesandcurrents.noaa.gov/).

| **Study area** | **Reference data collection method** | **Reference data spatial data type** | **Reference data classification type** | **Satellite imagery acquisition time** | **Satellite imagery view angle** | **Satellite imagery tidal height** |
| --- | --- | --- | --- | --- | --- | --- |
| Izembek Lagoon, AK | Satellite photointerpretation | Polygon | Presence | 14:12 AKDT | 14.0° | 1.00 |
| Padilla Bay, WA | Towed underwater camera | Point | Presence | 12:18 PDT | 24.5° | 2.17 |
| Elkhorn Slough, CA | Aerial photointerpretation | Polygon | Presence | 12:16 PDT | 10.8° | 1.61 |
| South Padre Island, TX | Field quadrats | Point | Percentage cover | 12:29 CDT | 13.5° | 0.40 |
| Tampa Bay, FL | Tropical Atlantic | Polygon | Percentage cover | 11:31 EST | 28.2° | -0.10 |
| Back Sound, NC | Aerial photointerpretation | Polygon | Percentage cover | 12:12 EDT | 21.7° | 0.24 |
| Mobjack Bay, VA | Aerial photointerpretation | Polygon | Percentage cover | 11:54 EDT | 27.6° | 0.56 |
| Tangier Sound, MD | Aerial photointerpretation | Polygon | Percentage cover | 12:17 EDT | 22.4° | 0.15 |
| Belmont Bay, VA | Satellite photointerpretation | Polygon | Percentage cover | 12:20 EDT | 18.2° | 0.26 |
| Broad Sound, MA | Aerial photointerpretation | Polygon | Presence | 11:37 EDT | 22.8° | 2.48 |
| Nahant Bay, MA | Aerial photointerpretation | Polygon | Presence | 11:37 EDT | 22.8° | 2.48 |

Table S3. Spectral characteristics of WorldView-2 and WorldView-3 in nanometers (nm). FWHM represents the full width half maximum range for each spectral band.

|  | **WorldView-2** | | **WorldView-3** | |
| --- | --- | --- | --- | --- |
| **Spectral band** | **FWHM** | **Center** | **FWHM** | **Center** |
| Coastal blue | 400-450 | 427 | 400-450 | 426 |
| Blue | 450-510 | 478 | 450-510 | 481 |
| Green | 510-580 | 546 | 510-580 | 547 |
| Yellow | 585-625 | 608 | 585-625 | 605 |
| Red | 630-690 | 659 | 630-690 | 661 |
| Red edge | 705-745 | 724 | 705-745 | 724 |
| NIR #1 | 770-895 | 833 | 770-895 | 832 |
| NIR #2 | 860-1040 | 949 | 860-1040 | 948 |

Table S4. Tidal station name and station ID used to retrieve tidal height measurements and predictions from the NOAA Tides & Currents database (https://tidesandcurrents.noaa.gov/).

| **Study area** | **Station name** | **Station ID** |
| --- | --- | --- |
| Izembek Lagoon, AK | Grand Point, Izembek Lagoon, AK | 9463058 |
| Padilla Bay, WA | Friday Harbor, WA | 9449880 |
| Elkhorn Slough, CA | Monterey, CA | 9413450 |
| South Padre Island, TX | South Padre Island Brazos Santiago Pass Station, TX | 8779750 |
| Tampa Bay, FL | Old Port Tampa, FL | 8726607 |
| Back Sound, NC | Beaufort, Duke Marine Lab, NC | 8656483 |
| Mobjack Bay, VA | Yorktown USCG Training Center, VA | 8637689 |
| Belmont Bay, VA | Dahlgren, VA | 8635027 |
| Tangier Sound, MD | Bishops Head, MD | 8571421 |
| Broad Sound, MA | Boston, MA | 8443970 |
| Nahant Bay, MA | Boston, MA | 8443970 |

Table S5. Region of interest (ROI) classes defined at each study area for image classification of WorldView-2 and WorldView-3 images via a deep convolutional neural network. Each study area contained four general classes, where applicable: seagrass, no seagrass, land, and no data. General classes of no seagrass, land, and no data were separated into sub-classes specific to each study area. Miscellaneous included non-seagrass substrates such as submerged rocks, benthic algae, and oyster reefs that differ spectrally from deep water and submerged sand. CDOM is chromophoric dissolved organic matter.

| **Study area** | **ROI classes** |
| --- | --- |
| Izembek Lagoon, AK | Seagrass, No seagrass (*deep water, submerged sand*), Land |
| Padilla Bay, WA | Seagrass, No seagrass (*deep water, submerged sand*), Land, No data (*turbid water, shadows*) |
| Elkhorn Slough, CA | Seagrass, No seagrass (*deep water, submerged sand, miscellaneous*), Land, No data (*CDOM, shadows*) |
| South Padre Island, TX | Seagrass, No seagrass (*deep water, submerged sand*)*,* Land |
| Tampa Bay, FL | Seagrass, No seagrass (*deep water, submerged sand*), Land (*land, intertidal*), No data (*CDOM*) |
| Back Sound, NC | Seagrass, No seagrass (*deep water, submerged sand*), Land, No data (*CDOM, turbid water*) |
| Mobjack Bay, VA | Seagrass, No seagrass (*deep water, submerged sand*), Land, No data (*CDOM, turbid water*) |
| Belmont Bay, VA | Seagrass, No seagrass (*deep water, submerged sand*), Land (*land, intertidal*), No data (*shadows*) |
| Tangier Sound, MD | Seagrass, No seagrass (*deep water, submerged sand*), Land, No data (*CDOM, turbid water*) |
| Broad Sound, MA | Seagrass, No seagrass (*submerged sand*), Land, No data (*CDOM*) |
| Nahant Bay, MA | Seagrass, No seagrass (*deep water, submerged sand*), Land |

Table S6. Ranges associated with reference data delineating seagrass percent cover and the average value used for comparison to satellite-derived seagrass percent cover.

| **Study area** | **Category** | **Lower bound** | **Upper bound** | **Average** |
| --- | --- | --- | --- | --- |
| Tampa Bay, FL | Patchy | 25% | 75% | 50% |
|  | Continuous | 75% | 100% | 87.5% |
| Back Sound, NC | Patchy | 5% | 70% | 37.5% |
|  | Continuous | 70% | 100% | 85% |
| Mobjack Bay, VA;  Tangier Sound, MD;  Belmont Bay, VA |  | 1% | 10% | 5.5% |
|  |  | 11% | 40% | 25.5% |
|  |  | 41% | 70% | 55.5% |
|  |  | 71% | 100% | 85.5% |

Table S7. Results of the post hoc pairwise Mann-Whitney U test and associated rank-biserial correlation (*r*_rb_) used to assess differences in satellite-indicated seagrass percent seagrass for increasing reference-indicated classes of seagrass percent cover; *n* is sample size.

| **Study area** | **Class_1_** | **Class_2_** | ***n*_1_** | ***n*_2_** | ***\|r*_rb_\|** | **Difference ^a^** |
| --- | --- | --- | --- | --- | --- | --- |
| Mobjack Bay, VA | 1% to10% | 11% to 40% | 16 | 33 | 0.07 | Negligible |
|  | 1% to10% | 41% to 70% | 16 | 30 | 0.08 | Negligible |
|  | 1% to 10% | 71% to 100% | 16 | 21 | 0.68 | Large |
|  | 11% to 40% | 41% to 70% | 33 | 30 | 0.01 | Negligible |
|  | 11% to 40% | 71% to 100% | 33 | 21 | 0.66 | Large |
|  | 41% to 70% | 71% to 100% | 30 | 21 | 0.64 | Large |
| Tangier Sound, MD | 1% to10% | 11% to 40% | 6 | 14 | 0.02 | Negligible |
|  | 1% to10% | 41% to 70% | 6 | 25 | 0.35 | Moderate |
|  | 1% to 10% | 71% to 100% | 6 | 12 | 0.56 | Large |
|  | 11% to 40% | 41% to 70% | 14 | 25 | 0.37 | Moderate |
|  | 11% to 40% | 71% to 100% | 14 | 12 | 0.52 | Large |
|  | 41% to 70% | 71% to 100% | 25 | 12 | 0.17 | Small |

^a^ Rank-biserial correlation was interpreted following Cohen (1988)


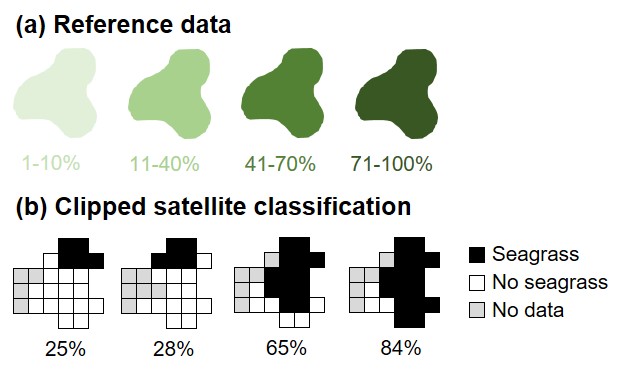


Figure S1. A conceptual diagram illustrating (a) reference-indicated and (b) satellite-indicated seagrass percentage cover. The satellite classification was clipped to the boundary of each individual reference polygon and percentage cover was computed as the percentage of satellite pixels classified as seagrass out of all valid satellite pixels, where valid satellite pixels were those classified as either seagrass or no seagrass.


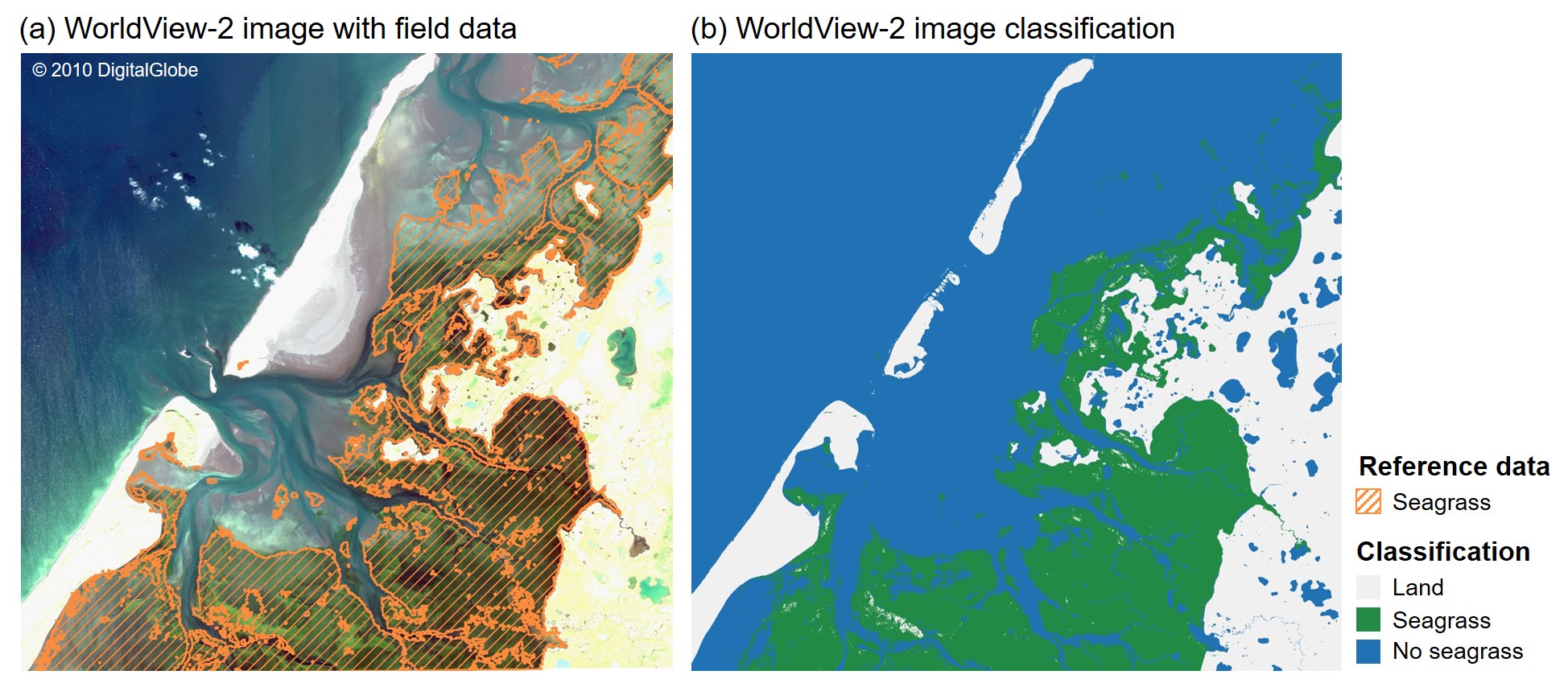


Figure S2. (a) A WorldView-2 satellite image acquired for Izembek Lagoon, AK, on 12 September 2018 overlaid with reference data delineating seagrass presence obtained from the Commission for Environmental Cooperation spanning 2002 through 2008, and (b) results of an image classification with classes for land, seagrass, and no seagrass.


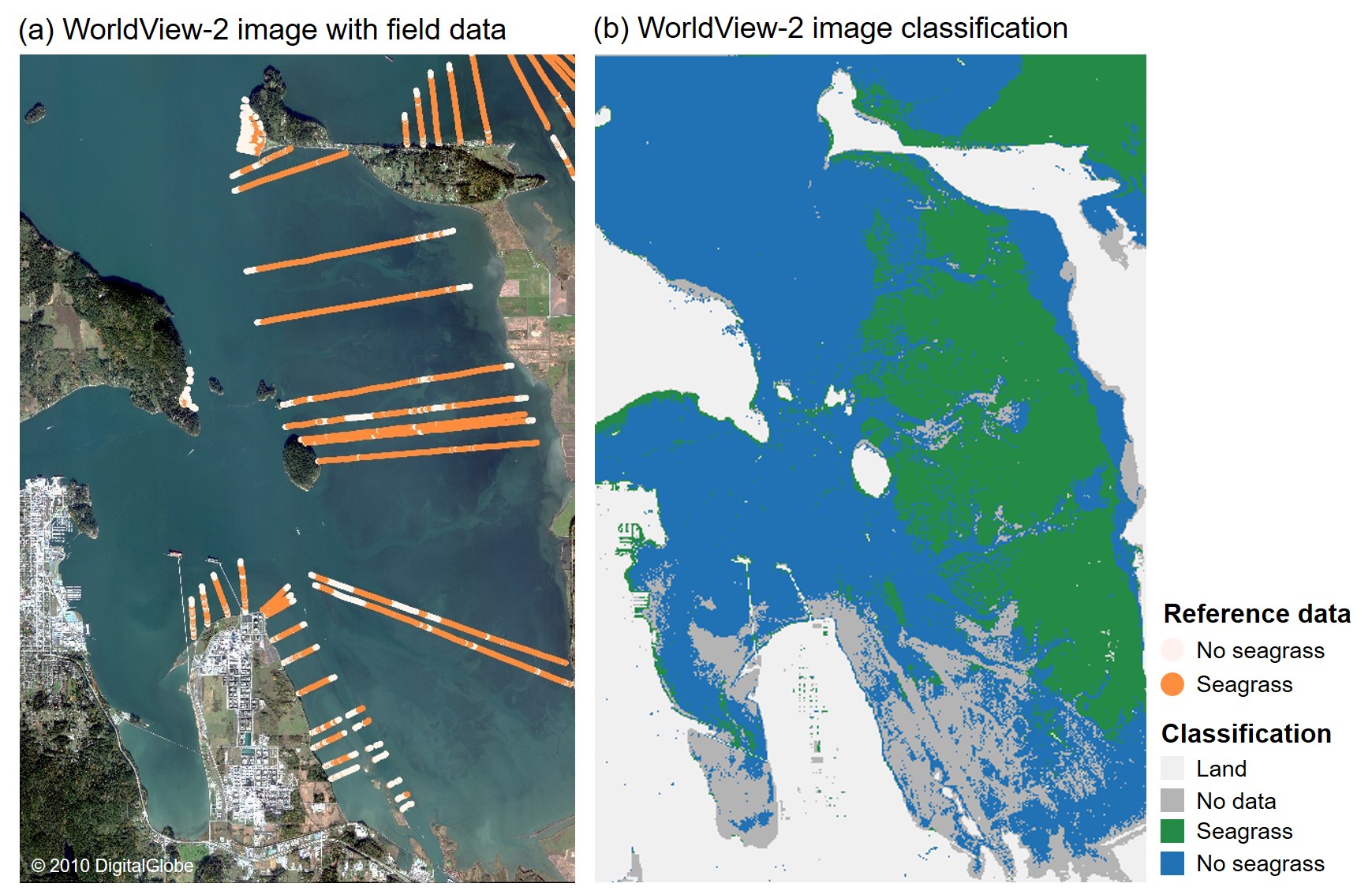


Figure S3. (a) A WorldView-2 satellite image acquired for Padilla Bay, WA, on 28 October 2017 overlaid with reference data delineating seagrass presence and absence obtained from the Washington State Department of Natural Resources in June and July 2017, and (b) results of an image classification with classes for land, no data, seagrass, and no seagrass.


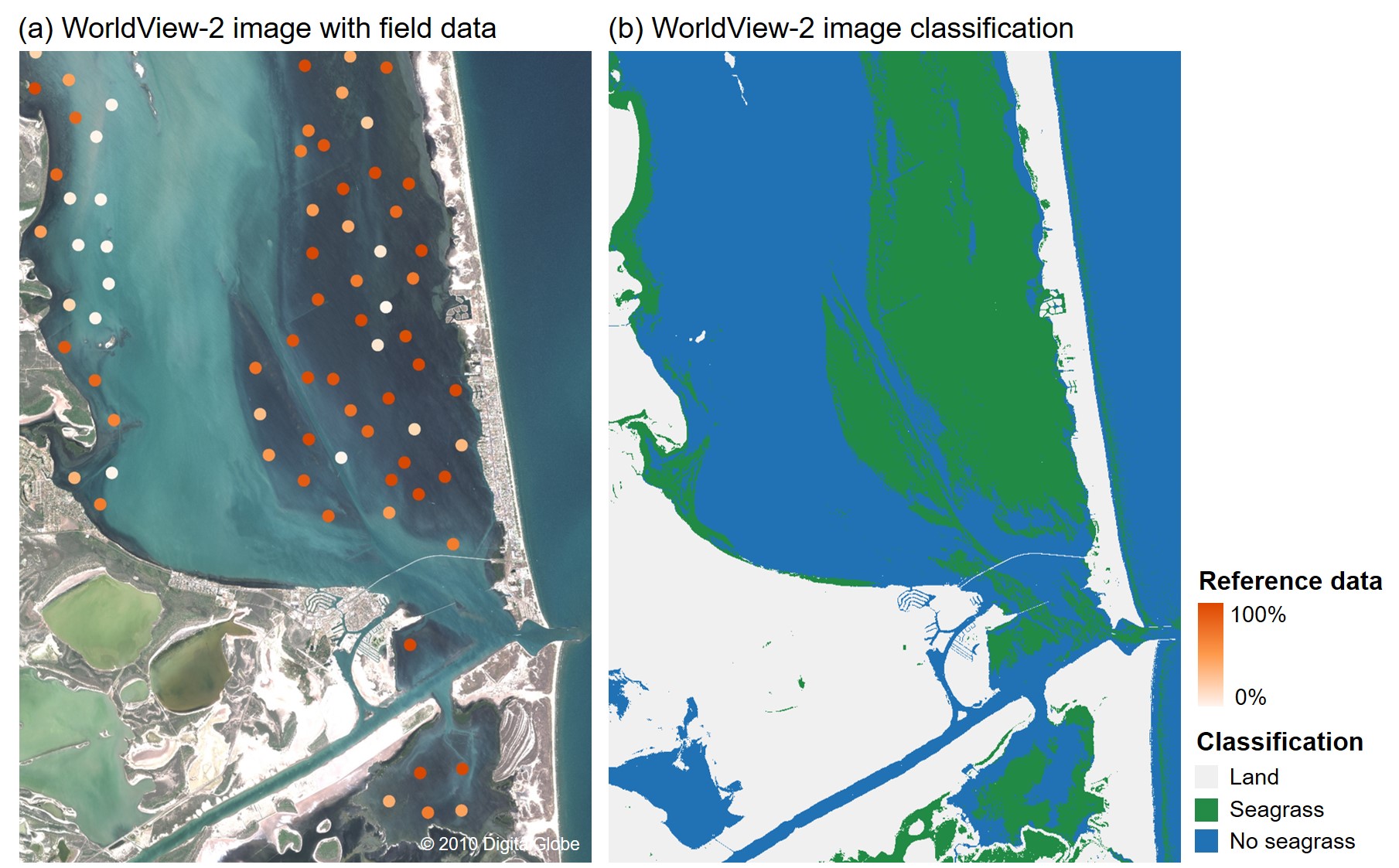


Figure S4. (a) A WorldView-2 satellite image acquired for South Padre Island, TX, on 1 August 2012 overlaid with reference data delineating seagrass percent cover obtained from the Texas Seagrass Monitoring Program in late summer through September 2012, and (b) results of an image classification with classes for land, seagrass, and no seagrass.


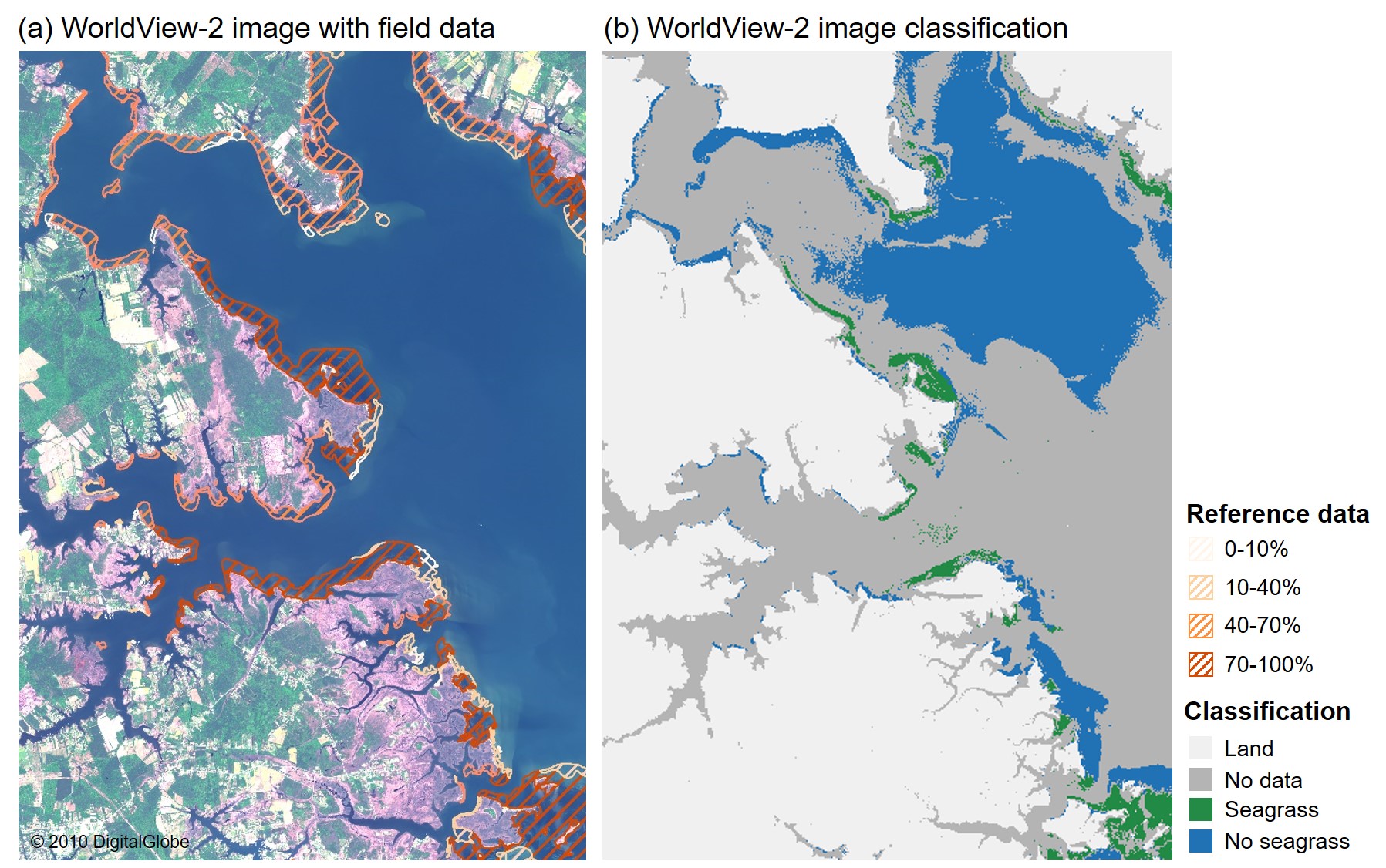


Figure S5. (a) A WorldView-2 satellite image acquired for Mobjack Bay, VA, on 4 May 2015 overlaid with reference data delineating seagrass percent cover obtained from the Virginia Institute of Marine Science in May through November 2015, and (b) results of an image classification with classes for land, no data, seagrass, and no seagrass.


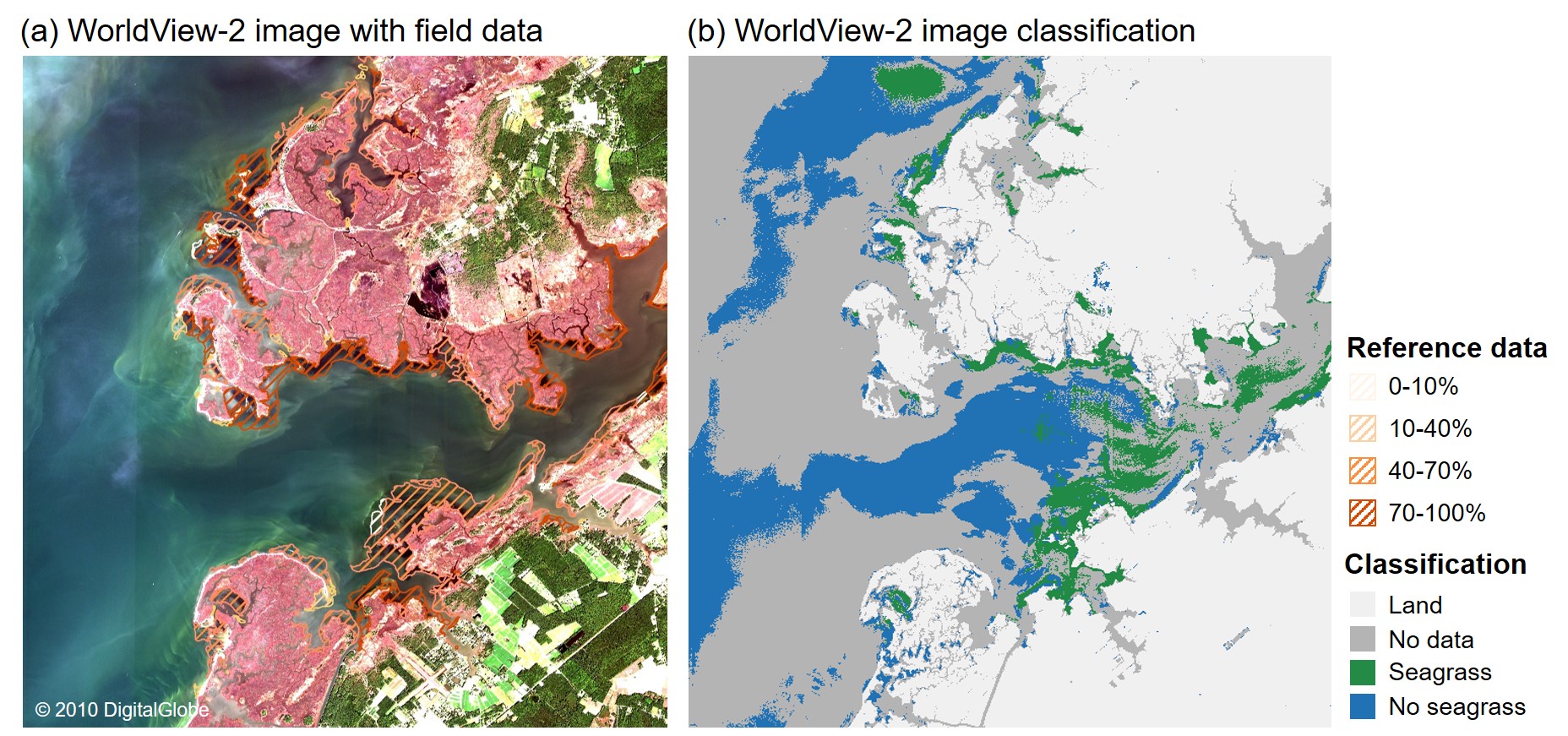


Figure S6. (a) A WorldView-2 satellite image acquired for Tangier Sound, MD, on 25 September 2017 overlaid with reference data delineating seagrass percent cover obtained from the Virginia Institute of Marine Science in May through November 2017, and (b) results of an image classification with classes for land, no data, seagrass, and no seagrass.


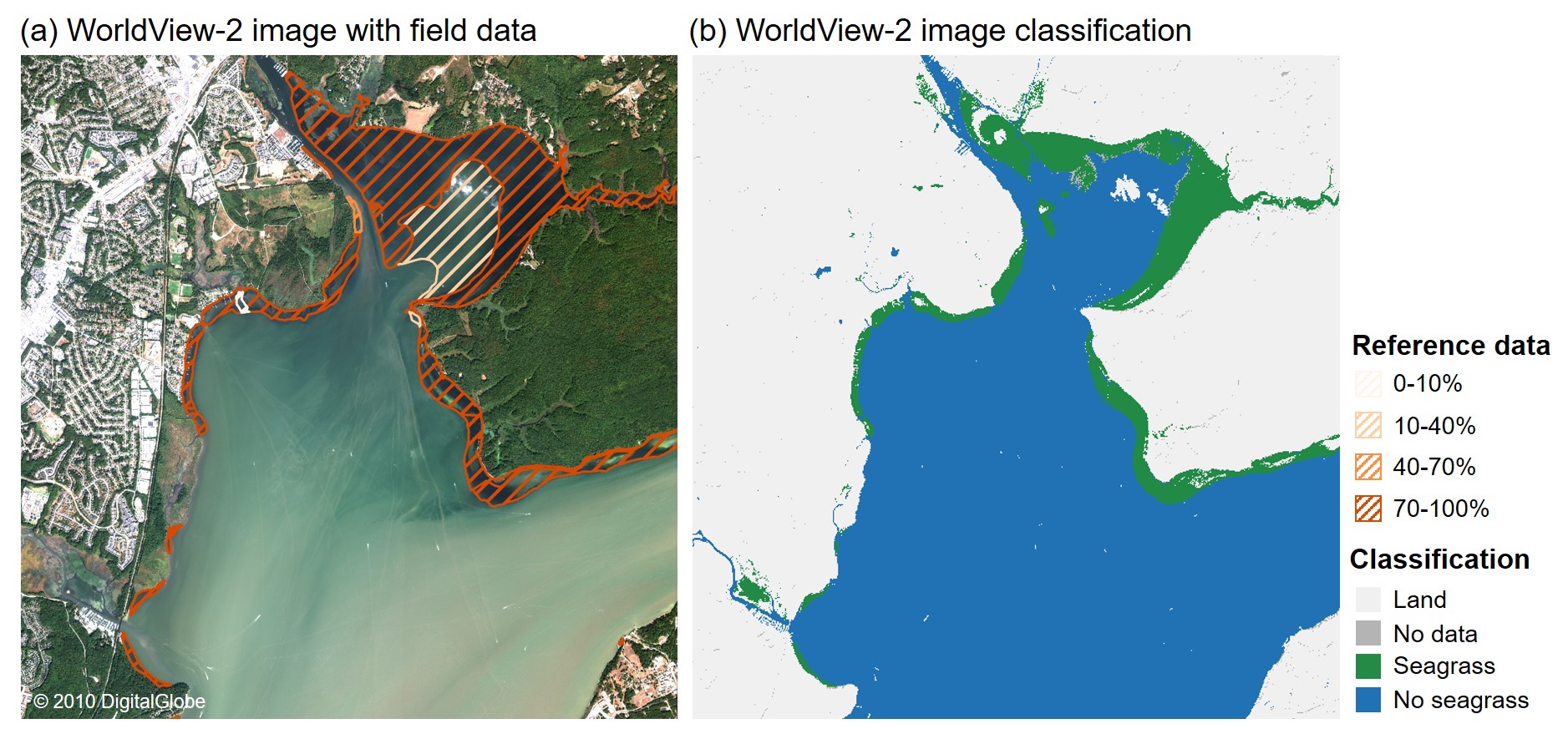


Figure S7. (a) A WorldView-2 satellite image acquired for Belmont Bay, VA, on 29 September 2019 overlaid with reference data delineating seagrass percent cover obtained from the Virginia Institute of Marine Science in summer 2019, and (b) results of an image classification with classes for land, seagrass, and no seagrass.


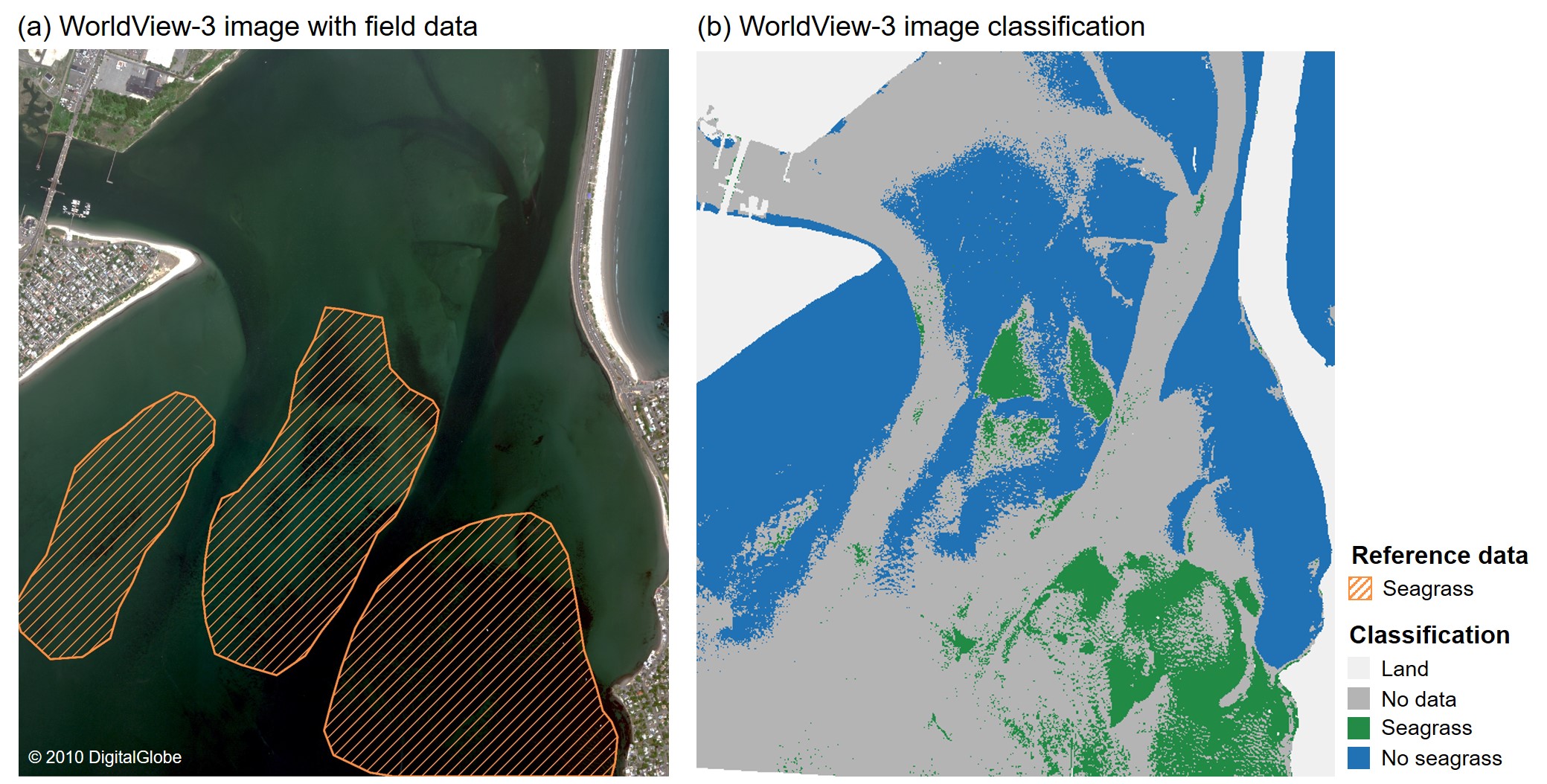


Figure S8. (a) A WorldView-3 satellite image acquired for Broad Sound, MA, on 7 June 2021 overlaid with reference data delineating seagrass presence obtained from the Massachusetts Department of Environmental Protection in 2016, and (b) results of an image classification with classes for land, no data, seagrass, and no seagrass.


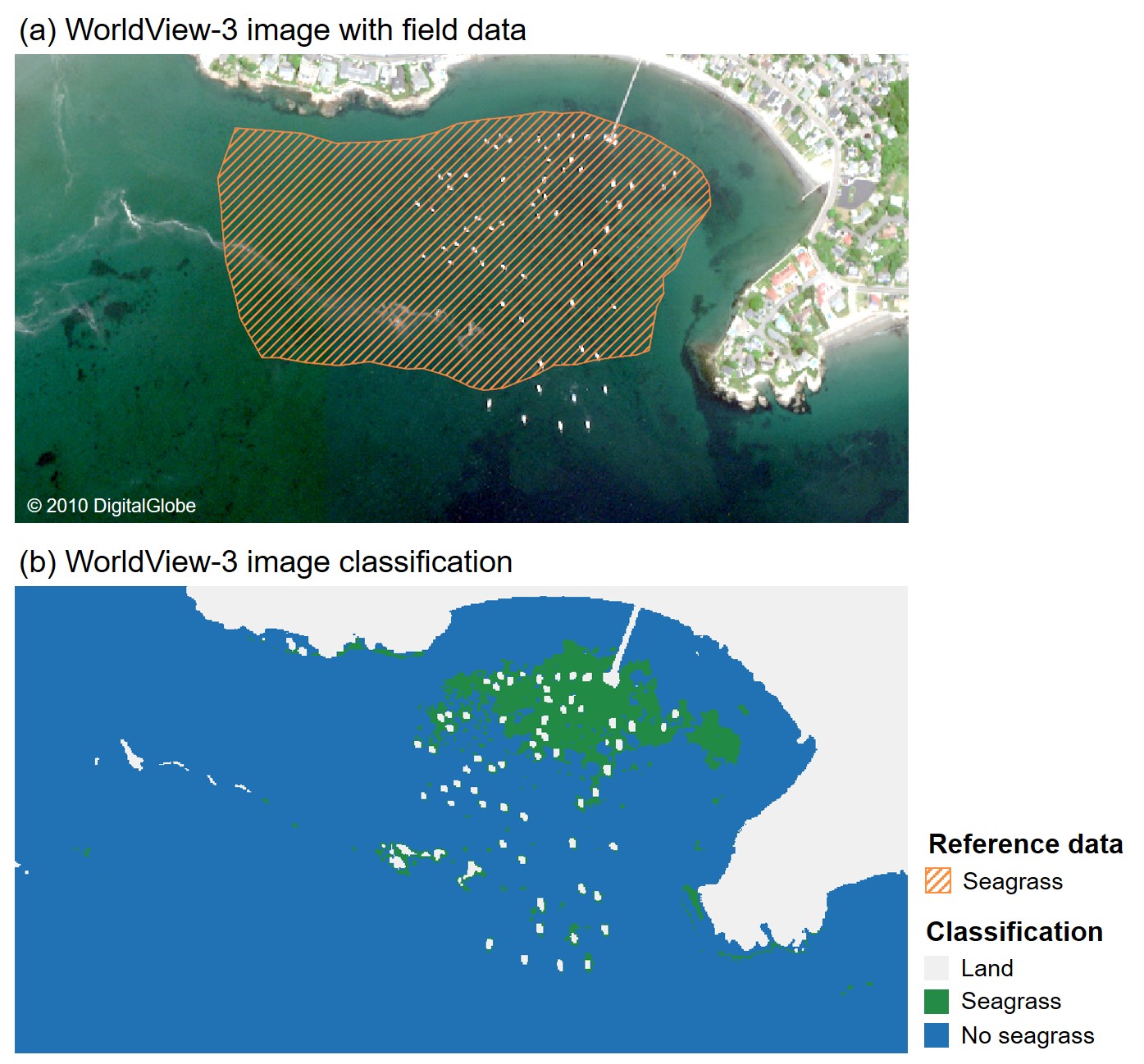


Figure S9. (a) A WorldView-3 satellite image acquired for Nahant Bay, MA, on 7 June 2021 overlaid with reference data delineating seagrass presence obtained from the Massachusetts Department of Environmental Protection in 2016, and (b) results of an image classification with classes for land, seagrass, and no seagrass.

**References**

Bartenfelder, A., Kenworthy, W.J., Puckett, B., Deaton, C., Jarvis, J.C., 2022. The Abundance and Persistence of Temperate and Tropical Seagrasses at Their Edge-of-Range in the Western Atlantic Ocean . Front. Mar. Sci. .

Carman, M.R., Colarusso, P.D., Neckles, H.A., Bologna, P., Caines, S., Davidson, J.D.P., Evans, N.T., Fox, S.E., Grunden, D.W., Hoffman, S., 2019. Biogeographical patterns of tunicates utilizing eelgrass as substrate in the western North Atlantic between 39^o^ and 47^o^ north latitude (New Jersey to Newfoundland). Manag. Biol. Invasions 10, 602. https://doi.org/10.3391/mbi.2019.10.4.02

Christiaen, B., Ferrier, L., Dowty, P., Gaeckle, J., Berry, H., 2019. Puget Sound Seagrass Monitoring Report: Monitoring Year 2016-2017. Nearshore Habitat Program. Washingt. State Dep. Nat. Resour. Olympia, WA.

Cohen, J., 1988. Set correlation and contingency tables. Appl. Psychol. Meas. 12, 425–434. https://doi.org/10.1177%2F014662168801200410

Dierssen, H.M., Bostrom, K.J., Chlus, A., Hammerstrom, K., Thompson, D.R., Lee, Z., 2019. Pushing the Limits of Seagrass Remote Sensing in the Turbid Waters of Elkhorn Slough, California. Remote Sens. . https://doi.org/10.3390/rs11141664

Erickson, A.A., Bell, S.S., Dawes, C.J., 2004. Does Mangrove Leaf Chemistry Help Explain Crab Herbivory Patterns? Biotropica 36, 333–343. https://doi.org/https://doi.org/10.1111/j.1744-7429.2004.tb00325.x

Hammerstrom, K., Grant, N., 2012. Assessment and monitoring of ecological characteristics of Zostera marina L beds in Elkhorn Slough, California. Elkhorn Slough Tech. Rep. Ser. 2012, 3.

Hogrefe, K.R., Ward, D.H., Donnelly, T.F., Dau, N., 2014. Establishing a baseline for regional scale monitoring of eelgrass (Zostera marina) habitat on the lower Alaska Peninsula. Remote Sens. 6, 12447–12477. https://doi.org/10.3390/rs61212447

Karl, T., Koss, W.J., 1984. Regional and National Monthly, Seasonal, and Annual Temperature Weighted by Area, 1895-1983, Historical Climatology Series 4-3. Asheville, NC.

Kuwayama, Y., Olmstead, S.M., Wietelman, D.C., Zheng, J., 2020. Trends in nutrient-related pollution as a source of potential water quality damages: A case study of Texas, USA. Sci. Total Environ. 724, 137962. https://doi.org/https://doi.org/10.1016/j.scitotenv.2020.137962

McPherson, B.F., Miller, R.L., 1994. Causes of light attenuation in Tampa Bay and Charlotte Harbor, southwestern Florida. JAWRA J. Am. Water Resour. Assoc. 30, 43–53.

Onuf, C.P., 2007. Laguna Madre. Handley, L., Altsman, D., DeMay, R., eds 1940–2002.

Orth, R.J., Marion, S.R., Moore, K.A., Wilcox, D.J., 2010. Eelgrass (Zostera marina L.) in the Chesapeake Bay region of mid-Atlantic coast of the USA: challenges in conservation and restoration. Estuaries and Coasts 33, 139–150. https://doi.org/10.1007/s12237-009-9234-0

PSEMP Marine Waters Workgroup, 2020. Puget Sound marine waters: 2019 overview.

Puget Sound Partnership, 2019. State of the Sound Report. Olympia, Washington.

Sherwood, E.T., Greening, H.S., Johansson, J.O.R., Kaufman, K., Raulerson, G.E., 2017. Tampa Bay (Florida, USA) documenting seagrass recovery since the 1980’s and reviewing the benefits. Southeast. Geogr. 57, 294–319. https://doi.org/10.1353/sgo.2017.0026

Short, F., Carruthers, T., Dennison, W., Waycott, M., 2007. Global seagrass distribution and diversity: a bioregional model. J. Exp. Mar. Bio. Ecol. 350, 3–20. https://doi.org/10.1016/j.jembe.2007.06.012

Taylor, E.J., Sowl, K.M., 2007. Izembek National Wildlife Refuge Biological Program Review-Final Report. Cold Bay, AK.

Testa, J.M., Lyubchich, V., Zhang, Q., 2019. Patterns and Trends in Secchi Disk Depth over Three Decades in the Chesapeake Bay Estuarine Complex. Estuaries and Coasts 42, 927–943. https://doi.org/10.1007/s12237-019-00547-9

Tunnell Jr., J.W., 2002. Geography, Climate, and Hydrography, in: Tunnell Jr., J.W., Judd, F.W. (Eds.), The Laguna Madre of Texas and Tamaulipas. Texas A&M University Press, College Station, Texas, pp. 7–27.

Turner, J.S., St-Laurent, P., Friedrichs, M.A.M., Friedrichs, C.T., 2021. Effects of reduced shoreline erosion on Chesapeake Bay water clarity. Sci. Total Environ. 769, 145157. https://doi.org/https://doi.org/10.1016/j.scitotenv.2021.145157

Ward, D.H., Markon, C.J., Douglas, D.C., 1997. Distribution and stability of eelgrass beds at Izembek Lagoon, Alaska. Aquat. Bot. 58, 229–240. https://doi.org/10.1016/S0304-3770(97)00037-5

Xiong, Y., Berger, C., 2010. Chesapeake Bay Tidal Characteristics. J. Water Resour. Prot. 2, 619–628. https://doi.org/10.4236/jwarp.2010.27071
